# Benzimidazole Resistance of Donkey Gastrointestinal Nematodes in Xinjiang, China, Assessed by the Faecal Egg Count Reduction Test and 18S r2b Amplicon Sequencing

**DOI:** 10.3390/vetsci13101104

**Published:** 2026-10-16

**Authors:** Min Yang, Sinan Qin, Yongqing Wang, Xinru Li, Mengjie Pi, Gulibostan Abla, Haorui Wang, Yang Zhang, Wei Zhang

**Affiliations:** 1 Parasitology Laboratory, College of Veterinary Medicine, Xinjiang Agricultural University, Urumqi 830052, China; 2 Xinjiang Key Laboratory of New Drug Research and Development for Herbivorous Animals (XJ-LNDRDHA), Urumqi 830052, China; 3 Xinjiang Regional Key Laboratory of Clinical Veterinary Medicine Research (XJRKLCVMR), Urumqi 830052, China

**Keywords:** anthelmintic resistance, benzimidazole, donkey, FECRT, 18S r2b, amplicon sequencing, strongyle

## Abstract

Gastrointestinal nematode (GIN) infection constrains equid production, and benzimidazole (BZ) resistance is widespread. Xinjiang is a major donkey-breeding region of China, yet anthelmintic-resistance data for donkeys are scarce. This study combined a faecal egg count reduction test (FECRT) with 18S r2b amplicon sequencing in female donkeys aged 1.0 and 1.5 years on one large-scale farm. Faecal samples from 74 animals were screened by the McMaster method; 30 with more than 150 eggs per gram (EPG) were enrolled and allocated to an albendazole-treated group (*n* = 15) and an untreated control group (*n* = 15), with counts on Days 0, 7 and 14. Overall, 63.51% (47/74) were excreting nematode eggs; strongyle-type eggs occurred in 62.16% (46/74) and *Parascaris* spp. eggs in 2.70% (2/74). The Day-14 group-level FECR was −24.49% (90% *CI* −93.56 to 19.93), far below the 99% target efficacy for benzimidazoles in equids, so the population is classified as resistant. No effect of treatment over time was detected when the control group was included in the models. Sequencing assigned almost all reads to a single genus, *Trichostrongylus*, and no cyathostomin genus was recovered; the 18S r2b marker resolves the strongyles of equids poorly, so these results are exploratory and species-level identification will require a different marker.

## Simple Summary

Gastrointestinal nematodes are a major constraint on donkey health, and benzimidazole drugs are widely used against them. We studied female donkeys aged 1.0 and 1.5 years on a large farm in Xinjiang, China, combining a faecal egg count reduction test, which estimates the efficacy of albendazole against the nematode population, with 18S r2b amplicon sequencing of nematode DNA recovered from the eggs. Of the 74 animals screened, 63.51% were excreting nematode eggs. Fourteen days after treatment the reduction was −24.49% (90% CI−93.56 to 19.93), far below the 99% target efficacy expected for benzimidazoles in equids, so the population on this farm is resistant. An untreated control group was analysed alongside the treated group, and no effect of treatment over time was detectable. Sequencing assigned almost all reads to a single genus, *Trichostrongylus*, and no cyathostomin genus was recovered; because the marker resolves the strongyles of equids poorly, these results are exploratory.

## 1. Introduction

Gastrointestinal nematode (GIN) infections are a major constraint on equid health and production worldwide [1,2]. These infections cause growth retardation, diarrhoea and weight loss, and clinical signs are more pronounced in young animals [3,4]. Control still depends heavily on anthelmintics, and the benzimidazoles (BZs) remain among the most widely used because of their broad spectrum, high efficacy and low toxicity [5]. However, prolonged and frequent use has led to increasing BZ resistance in livestock nematodes [6], and the reliable detection of resistance is therefore essential for sustainable control.

The faecal egg count reduction test (FECRT) is the accepted field method for detecting anthelmintic resistance in equids [7,8]. It is simple and inexpensive, but it does not identify nematode species, and equine strongylid eggs cannot be reliably assigned to genus by light microscopy [9]. Amplicon sequencing of nematode DNA recovered from eggs can resolve community composition and relative abundance and therefore complements the FECRT [10,11]. The 18S r2b region (~400 bp) is a nematode-specific marker that has been used for genus-level profiling of equine strongylids, and paired-end 250 bp Illumina sequencing (PE250) provides the read length required for this amplicon. We note at the outset that ITS-2 gives better species-level resolution for cyathostomins than 18S r2b [12]; 18S r2b was chosen here for its broad nematode coverage.

BZ resistance in equid GINs has been reported from many regions. In Norway, it was already prevalent in equine small strongyles [13]; in France, reduced efficacy of the major anthelmintics against horse cyathostomins has been documented [14]; in Ethiopia, ivermectin remained effective in working donkeys whereas suspected BZ resistance was detected [15]; in Romania, fenbendazole cleared horses but not donkeys [16]; and in Iran, no BZ resistance was found in Kermanshah Province [17]. Reviews have repeatedly emphasised how sparse the resistance data for donkeys are [18,19], and the same is true for Xinjiang [20,21,22]. Because resistance management depends on preserving refugia and avoiding whole-group treatment [23,24], regional surveillance data of the kind reported here have direct practical value. Field surveys of working donkeys continue to report a high burden of strongyle infection alongside variable drug efficacy [25], and in Europe, *Strongylus vulgaris* has been found to be more common in donkeys than previously assumed [26].

This study therefore combined a WAAVP-based FECRT with 18S r2b amplicon sequencing in female donkeys aged 1.0 and 1.5 years on a single large-scale farm in the Nanshan area of Urumqi, Xinjiang. The objectives were (i) to determine the level of BZ resistance in the GIN community of these donkeys, (ii) to describe the composition and longitudinal dynamics of the nematode community recovered from eggs before and after treatment, and (iii) to provide baseline data for resistance surveillance on the farm. The statistical analysis of the sequencing data follows the repeated-measures structure of the design, with the untreated control group included in every formal analysis.

## 2. Materials and Methods

### 2.1. Study Area and Animals

The study was carried out at a large-scale donkey farm in the Nanshan area of Urumqi, Xinjiang, China (86°37′–88°58′ E, 42°45′–44°08′ N), between 16 December 2025 and 4 January 2026. The area lies at 1600–2200 m with a mid-temperate, semi-arid mountain climate; a winter temperature inversion creates a warm belt 4–5 °C warmer than the urban area, which may favour the year-round survival of free-living nematode stages [27]. The farm kept approximately 300 donkeys. Only female animals of the Xinjiang donkey breed and its crosses were used, from two age strata of 1.0 year and 1.5 years (15 animals each), so that age and sex were controlled. Management was semi-confined with deworming every six months; the last treatment before enrolment was about six months earlier and used albendazole. Animal welfare ethics approval was obtained from the Animal Welfare and Ethics Committee of Xinjiang Agricultural University, and the farm owner gave written permission for animal use and sample collection.

### 2.2. Epidemiological Screening, Enrolment and Faecal Examination

Faecal samples from 74 female donkeys were examined by the McMaster technique and eggs were classified morphologically using standard keys [9]. Animals with EPG > 150 were eligible. The 30 enrolled animals were stratified by age (1.0 year, *n* = 15; 1.5 years, *n* = 15) and by body weight, then allocated within each stratum by sealed-envelope randomisation to a treated and an untreated control group, with 15 animals each. The groups did not differ in age structure (eight animals aged 1.0 year and seven aged 1.5 years in each) and both spanned the permitted weight range of 100–130 kg (treated 114.0 ± 11.2 kg, control 115.3 ± 11.9 kg; mean ±*SD*). Baseline egg counts did not differ (median 400 versus 350 EPG; *Mann–Whitney U*, *p* = 0.098).

Samples were collected in the morning on Day 0 (before treatment), Day 7 and Day 14. Faeces were taken directly from the rectum or from freshly voided material within 5 min of defecation, kept in cool boxes at 4 °C and processed within 2 h of arrival. Egg counts were performed in a modified McMaster chamber with two chambers totalling 0.6 mL; 2 g of faeces were suspended in 58 mL of saturated saline (1:30 dilution), the suspension was filtered through a 150 µm sieve, and both chambers were counted at 40× magnification by a single operator blinded to group allocation. Duplicate counts agreed within 10% for all samples. Counts were multiplied by 100, so one egg seen in a chamber corresponds to 100 EPG.

The multiplication factor of 100 in Equation (2) follows from the 1:30 dilution; because the FECR is a ratio of egg counts it cancels, and it does not affect any result reported here.

Infection rate (%) = (number of positive samples/total samples) × 100(1)

EPG = (eggs in left chamber + eggs in right chamber) × 100(2)

### 2.3. Treatment Protocol

Animals in the treatment group received a single oral dose of albendazole (Zhucheng Mubao Veterinary Drug Co., Ltd., Zhucheng, China; 50 mg per tablet; Chinese Veterinary Drug Approval No. 070131195) at 10 mg/kg body weight, which is the upper end of the labelled equine range and the dose recommended by the 2023 guideline for equids [28]. Because every animal weighed a multiple of 5 kg and the product is supplied as 50 mg tablets, the target dose always corresponded to a whole number of tablets (20 at 100 kg, 22 at 110 kg, 24 at 120 kg, 26 at 130 kg), so the administered dose was exactly 10.0 mg/kg for all 15 treated animals, with no rounding (Table 1). Tablets were given orally by hand after the mouth had been checked for feed residues, and swallowing was confirmed by observation. No anthelmintic was given to the control group.

### 2.4. Faecal Egg Count Reduction and Resistance Classification

Calculation and interpretation followed the 2023 WAAVP guideline for the FECRT in equids [28]. The guideline recommends a paired design based on pre- and post-treatment counts from the same animals; because an untreated control group was also maintained, both the paired and the unpaired estimate are reported. The unpaired estimate is taken as primary. Classification follows Box 5: target efficacy 99% and a research-protocol lower efficacy target of 95%, with the grey zone between them, classification by the 90% confidence interval, and resistance declared when the upper 90% confidence limit falls below 99%.

Paired design (Equation (3)):

FECR (%) = 100 × (1 − mean EPG of the treated group on Day 14/mean EPG of the treated group on Day 0)(3)

Unpaired design (Equation (4)):

FECR (%) = 100 × (1 − mean EPG of the treated group on Day 14/mean EPG of the control group on Day 14)(4)

Confidence limits were obtained with the log-scale method used for FECRT data, in which the variance is that of the ratio of the two arithmetic means and the exponential factor is applied to that ratio rather than to the reduction itself [17,28]:

V^2^ = (S_T^2^/(n_T × x_T^2^)) + (S_C^2^/(n_C × x_C^2^)) (5)

UCL = 100 × [1 − (x_T/x_C) × exp(−t_(1−α/2) × √V^2^)] (6)

LCL = 100 × [1 − (x_T/x_C) × exp(+t_(1−α/2) × √V^2^)] (7)

where x_T and x_C are the arithmetic means of the treated and control groups on Day 14, S_T^2^ and S_C^2^ are the corresponding sample variances, n_T = n_C = 15, and t is the critical value of the t distribution with *df* = n_T + n_C − 2 = 28. As required by the 2023 guideline, a 90% confidence interval was used (α = 0.10, t_(0.95,28) = 1.701), because the classification rests on two one-tailed tests.

Classification thresholds for cyathostomins in equids treated with benzimidazoles follow Box 5 of the 2023 guideline [28]: the target (expected) efficacy is 99%, the lower efficacy target for a research protocol is 95% (grey zone 95–99%), and classification uses the 90% confidence interval. The population is classified as resistant when the upper 90% confidence limit is below 99%, as susceptible when the lower 90% limit is at or above 95% and the upper limit is at or above 99%, and as inconclusive otherwise. Individual animal FECRs are reported for descriptive purposes only, and the efficacy estimate is the group-level FECR.

The guideline further requires, for a research protocol at this egg count magnitude, a mean of at least eight eggs counted in pre-treatment samples (or at least 280 eggs in total when only seven animals are used) [28]. As reported in Section 3.3, the present dataset does not satisfy that requirement at either candidate multiplication factor; this is stated as a limitation on the precision of the efficacy estimate.

### 2.5. DNA Extraction, PCR Amplification and Sequencing

Eggs from the Day 0, Day 7 and Day 14 samples were concentrated, pooled into 20 mL tubes, labelled and sent to a commercial provider for PE250 amplicon sequencing of the 18S r2b region. Samples were selected on the basis of the egg counts: material was sent wherever the count was non-zero, with a zero count being taken to indicate that no eggs were present. That decision, taken before sequencing, determined which animals could enter the molecular analysis. Of the 90 samples the design could have produced (30 animals × 3 time points), 80 were sent and returned and 63 yielded usable data after removal of low-abundance OTUs; all 63 came from animals whose count was non-zero at every time point. Twenty-two animals (15 control, seven treated) met that condition and three control samples were then lost to low read numbers, so Section 3.4, Section 3.5, Section 3.6, Section 3.7 and Section 3.8 rest on 63 samples from 21 animals (14 control, seven treated). Genomic DNA was extracted with the Mag Beads FastDNA Kit for Soil (MP Biomedicals, CA, USA), checked by 1% agarose gel electrophoresis, quantified on a Nanodrop spectrophotometer and stored at −20 °C. Per-sample DNA concentrations were not returned by the provider; a column is reserved in Table S10.

The 18S r2b region (~400 bp) was amplified with the nematode-specific primers NF1 (5′-GGTGGTGCATGGCCGTTCTTAGTT-3′) and 18Sr2b (5′-TACAAAGGGCAGGGACGTAAT-3′) [29]. Reactions were performed in 25 μL containing 12.5 μL of 2× Taq Master Mix, 0.5 μM of each primer, 2 μL of template DNA and nuclease-free water to volume, with the following programme: 95 °C for 3 min; 35 cycles of 95 °C for 30 s, 55 °C for 30 s and 72 °C for 30 s; and 72 °C for 5 min. Each run included a no-template negative control and a positive control consisting of genomic DNA from a confirmed strongyle-positive sample, and all reactions were performed in triplicate. Products were purified, quantified, pooled into libraries and sequenced on the Illumina platform in PE250 mode [30].

### 2.6. Bioinformatics

Reads were processed in QIIME 2 v2024.5 [31] with the VSEARCH plugin [32]. Primers were trimmed allowing a maximum of 2 bp mismatches; reads were merged with a minimum overlap of 20 bp; quality filtering used a maximum expected error of 1.0; and chimaeras were removed with UCHIME. OTUs were clustered at 97% similarity [33]. Taxonomy was assigned against the PR2 database v4.14.0 [34] using a naive Bayes classifier with a confidence threshold of 0.7. Sequences annotated as *Austrostrongylus victoriensis* were removed, and only sequences assigned to *Trichostrongylus*, *Parascaris* and *Coronocyclus* were retained; low-abundance OTUs (fewer than 10 reads in total) were discarded. The resulting OTU table contained 788 OTUs across 63 samples and was rarefied to 2120 reads per sample for all diversity analyses. The taxonomic profiles before and after filtering are given in Supplementary Table S4.

### 2.7. Statistical Analysis and Software

The design is a two-group (treated, control) by three-time-point (Days 0, 7 and 14) repeated-measures design with the animal as the experimental unit. The untreated control group was included in every formal analysis. All analyses were performed in Python 3.12 (NumPy 2.3, pandas 3.0, SciPy 1.18, statsmodels 0.15) and cross-checked in R 4.3.3 [35].

Faecal egg counts and FECRT. Group means, standard deviations and the FECR were computed with NumPy2.3/pandas3.0 and confidence limits from Equations (5)–(7) with the t distribution (SciPy). Settings for reproducibility: arithmetic group means; unbiased sample variances with n−1 (NumPy ddof = 1); t(0.95, 28) = 1.7011 and t(0.975, 28) = 2.0484 for the 90% and 95% intervals, from scipy.stats.t.ppf with df = 28; the multiplication factor cancels in the ratio. Baseline comparability used Mann–Whitney U and, for comparison, Welch and Student’s *t* tests; within-group change used the Friedman test (tie-corrected Kendall) and the paired Day 0 versus Day 14 comparison used the Wilcoxon signed-rank test. Group, time and their interaction were evaluated with a negative-binomial generalised linear model with animal-clustered robust standard errors, with a linear mixed model on log(EPG + 1) as sensitivity analysis. Individual FECR values are descriptive only.

Alpha diversity. Chao1, observed OTUs, Shannon, Simpson and Pielou evenness were computed with NumPy on the OTU table rarefied to 2120 reads per sample, and Good’s coverage was computed as a completeness check. Each index was modelled as index ~ group × day with animal as a random intercept (REML, statsmodels), and the group × day interaction was tested with a Wald test. Friedman tests within each group and Wilcoxon signed-rank tests with Holm correction served as repeated-measures sensitivity analyses.

Beta diversity. Aitchison distances were computed from centre-log-ratio (CLR) transformed genus counts with multiplicative replacement of zeros (0 → 0.5 × the smallest positive count), and Bray–Curtis distances from the untransformed counts (NumPy). PERMANOVA (9999 permutations) tested group, day and the group × day interaction; permutations were restricted within animal for the within-animal factor (day), whereas unrestricted permutations were used for the between-animal factor (group), for which restriction within animal is not valid because group is constant within animal. PERMDISP (9999 permutations) tested the homogeneity of multivariate dispersion, and principal coordinates analysis was used for ordination. All permutation tests were implemented in NumPy with a fixed seed (20260101) to ensure reproducibility, and the implementation was validated against hand-computed values.

Genus-level composition. Genus counts were CLR-transformed after multiplicative zero replacement and each genus modelled as CLR ~ group × day with animal as a random intercept (REML), with Benjamini–Hochberg adjustment across genera. Because the samples are not independent, all genus-level comparisons used this mixed model and its Friedman companion test; no test assuming independent samples was applied.

Prevalence. Infection rates were reported with 95% confidence intervals computed by the Wilson score method and, for comparison, by the Clopper–Pearson exact method (SciPy); the Wald interval was not used.

Verification of the taxonomic assignments. Because the 18S r2b region resolves equine strongylids poorly [12], the classifier output was checked independently against GenBank. A reference set of nematode 18S sequences was retrieved by organism query (Strongylida, Trichostrongylidae, *Trichostrongylus*, Cyathostominae, *Cyathostomum*, *Coronocyclus*, *Cylicocyclus*, *Cylicostephanus*, *Poteriostomum*, *Triodontophorus*, *Parascaris*, *Strongylus*, *Austrostrongylus*, *Haemonchus*, *Cooperia*, *Ostertagia*, *Teladorsagia*), filtered to at least 200 bp, and searched with the 788 representative OTUs using BLASTN 2.17.0 in megablast mode (max_target_seqs 5, E-value 1 × 10^−20^); per-OTU results are in Table S9. For each OTU, the best hit within each candidate genus was compared to the best hit overall, and the bitscore gap between the leading and second genus measured how far the marker separates them.

## 3. Results

### 3.1. Egg Morphology and Infection Rate in 74 Female Donkeys

**Figure 1:**
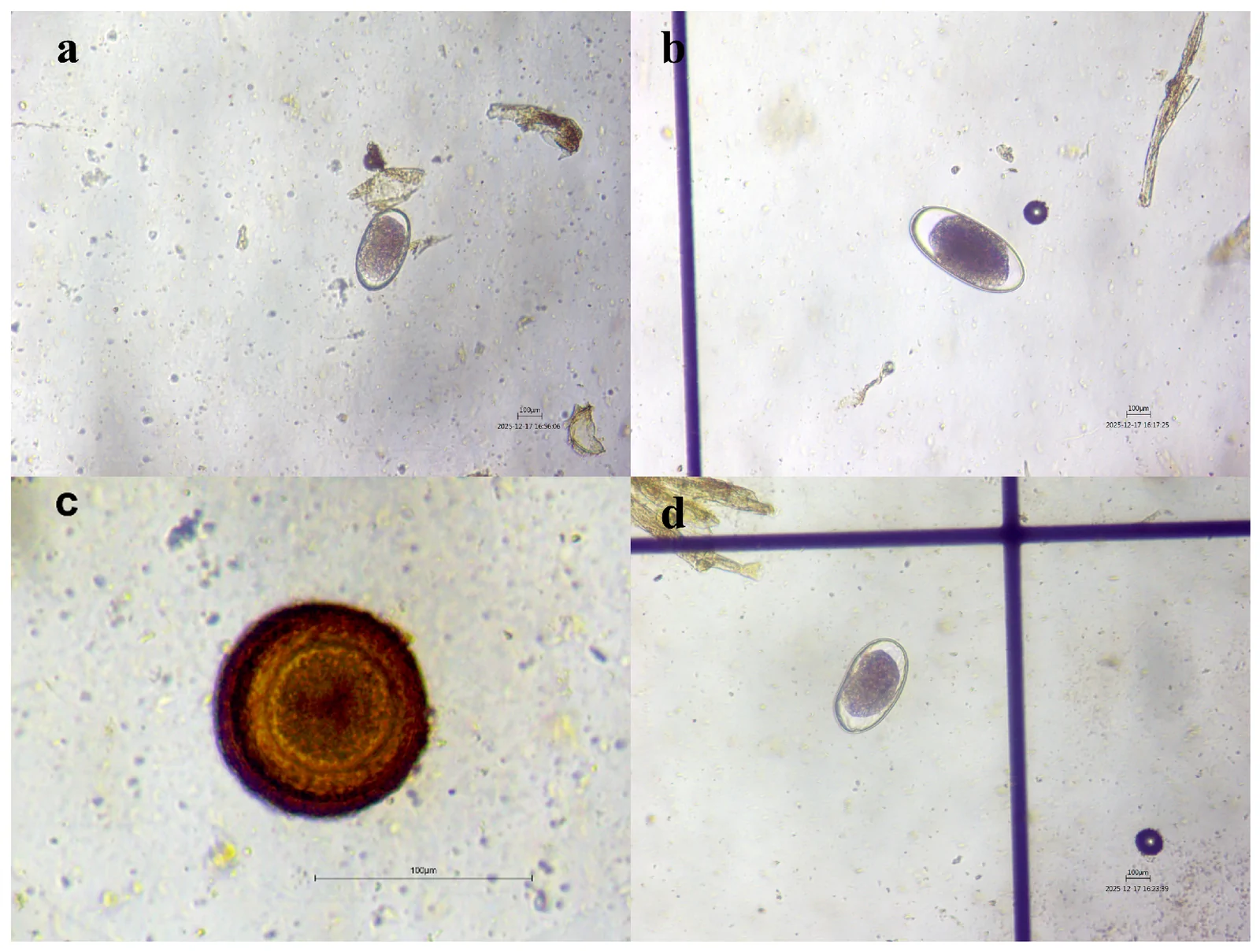
Morphological characteristics of the gastrointestinal nematode eggs recovered from donkey faeces. (**a**,**b**,**d**) Strongyle-type eggs; (**c**) *Parascaris* spp. egg. Scale bars = 100 µm. The micrographs are supplied by the authors. Panel (**c**) shows a *Parascaris* egg.

Of the 74 female donkeys examined, 47 were excreting nematode eggs, which is a positivity rate of 63.51% (47/74). Each morphotype was counted independently, so samples containing both contributed to both categories. Strongyle-type eggs were found in 46 samples (45 single infections plus one mixed), 62.16% (46/74; Wilson 95% *CI* 50.77–72.35%), with EPG values from 100 to 1500. *Parascaris* spp. eggs were found in two samples, 2.70% (2/74; 95% *CI* 0.74–9.33%), with an EPG of 100. One sample contained both morphotypes, 1.35% (1/74; 95% CI 0.24–7.27%); the EPG of 400 recorded for it is the combined total, not the value for one morphotype (Table 2 and Figure 1). These estimates describe the 74 animals on this farm and cannot be extrapolated.

### 3.2. Egg Counts Before and After Treatment

**Figure 2:**
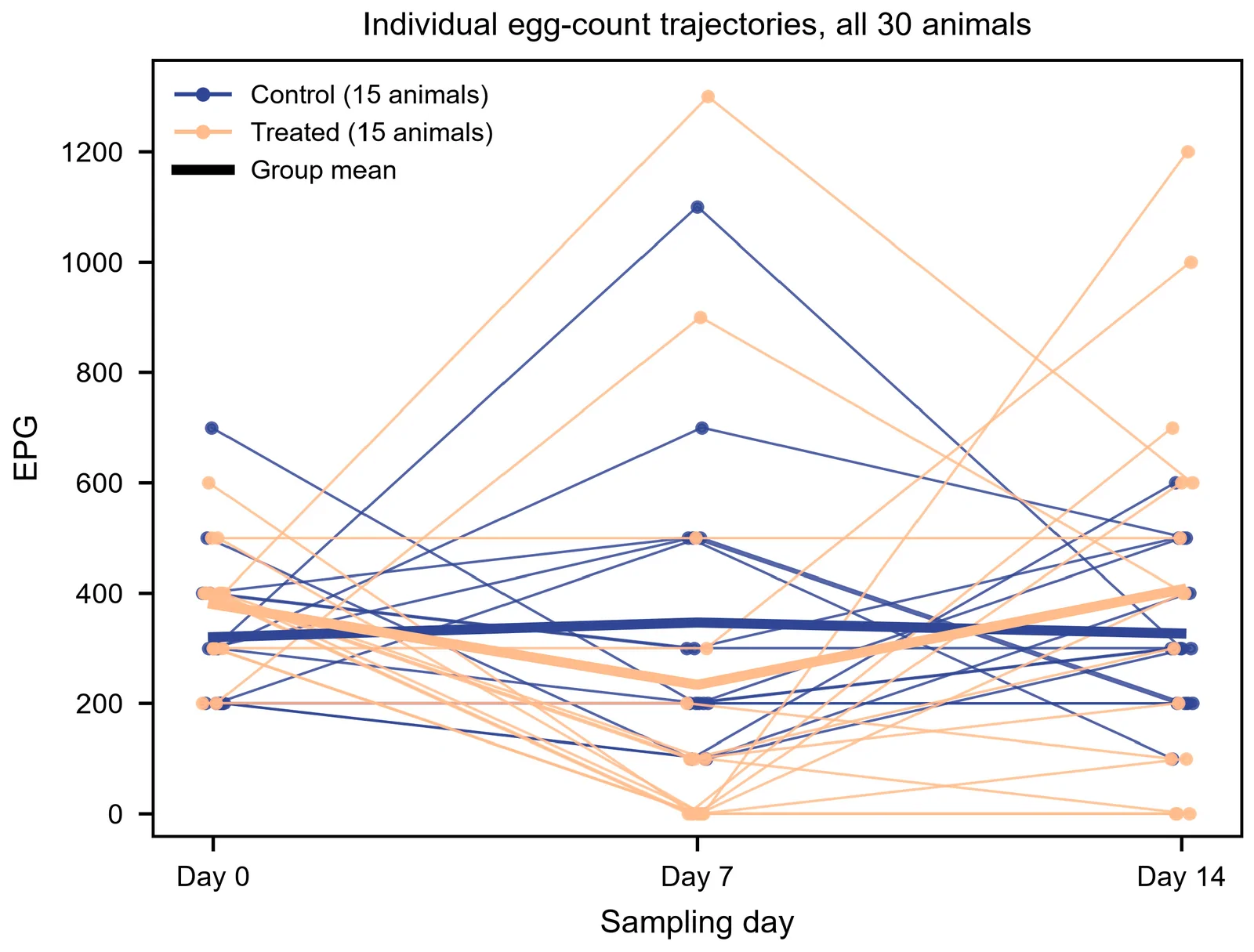
Individual faecal egg count (EPG) trajectories for all 30 enrolled animals on a single shared axis: 15 albendazole-treated and 15 untreated control animals. A small horizontal offset is applied at each time point so that coincident individuals remain distinguishable. The thick lines give the arithmetic group means. Counts were obtained by the McMaster method on Days 0, 7 and 14. The group × time interaction of the negative-binomial model including both groups was not significant (*p* = 0.873, *n.s.*). Significance convention: *n.s.*, *p* ≥ 0.05; *p* < 0.05; *p* < 0.01; *p* < 0.001; *Mann–Whitney* U test.

On Day 0, the mean EPG was 380.0 ± 108.2 in the albendazole-treated group (*n* = 15) and 320.0 ± 142.4 in the untreated control group (*n* = 15); the groups did not differ significantly at baseline (*Mann–Whitney U*, *p* = 0.098; Supplementary Table S1). Individual trajectories are shown in Figure 2 and listed in Supplementary Table S1. In the treated group, the mean EPG was 233.3 on Day 7 and 406.7 ± 367.4 on Day 14; in the control group, the corresponding values were 346.7 and 326.7 ± 143.8. Individual Day 14 FECR values in the treated group ranged from −233.3% to 100.0%, and in the control group from −100.0% to 75.0%; the distributions did not differ between groups (*Mann–Whitney U*, *p* = 0.851). Individual reductions are presented descriptively only, because averaging per-animal percentages is not a valid estimator of group efficacy.

No change in egg counts over time was detected within either group (*Friedman* test: treated χ^2^ = 4.308, *p* = 0.116; control χ^2^ = 0.875, *p* = 0.646), and the paired Day 0 versus Day 14 comparison was likewise not significant (Wilcoxon signed-rank: treated *p* = 0.925, control *p* = 0.650). The negative-binomial model including both groups found no significant group × time interaction (χ^2^ = 0.026, *p* = 0.872); a linear mixed model on log(EPG + 1) gave the same conclusion (χ^2^ = 1.395, *p* = 0.238). Group and time main effects were also not significant in either model (*p* > 0.37 and *p* > 0.88, respectively).

### 3.3. Faecal Egg Count Reduction and Resistance Classification

**Figure 3:**
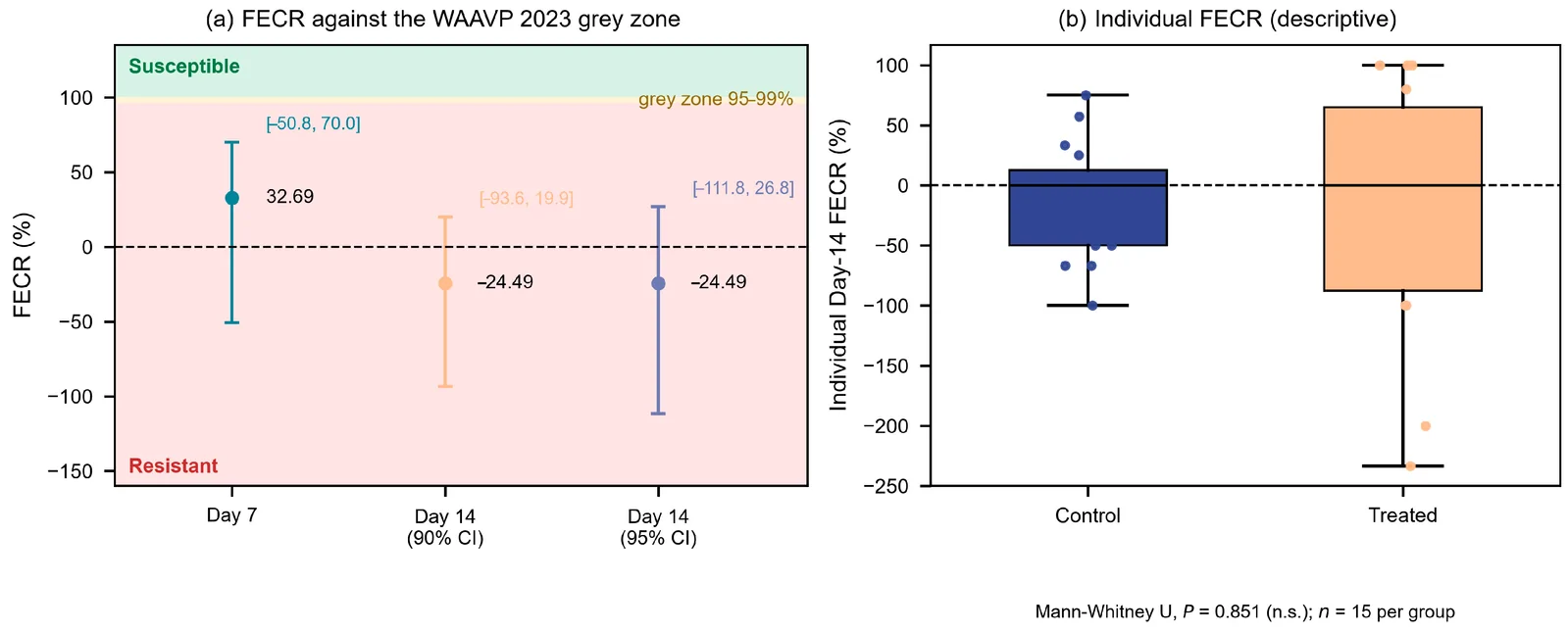
Faecal egg count reduction and resistance classification. (**a**) FECR point estimates with 90% and, for comparison, 95% confidence limits against the grey zone defined by the 2023 WAAVP guideline for cyathostomins treated with benzimidazoles (target efficacy 99%; research-protocol grey zone 95–99%); the population is classified as resistant because the upper 90% confidence limit lies below 99%. (**b**) Individual Day 14 FECR values, shown descriptively; the difference between treated and untreated animals was not significant (*Mann–Whitney U*, *p* = 0.851, *n.s.*).

The Day 14 group-level FECR computed against the untreated control group (Equation (4)) was −24.49% with a 90% confidence interval of −93.56% to 19.93% (*t* = 1.701, *df* = 28); the corresponding 95% interval is −111.81% to 26.83%. The paired FECR from the same animals was −7.02% and the geometric-mean paired FECR was 67.39%. At Day 7, the FECR was 32.69% (90% *CI* −50.83% to 69.96%). Because the upper 90% confidence limit of the Day 14 FECR lies well below the 99% target efficacy for benzimidazoles against cyathostomins in equids [28], the population is classified as resistant to albendazole (Table 3, Figure 3).

### 3.4. Community Composition and the Effect of the Taxonomy Filter

Amplicon sequencing produced usable data for 63 samples: 42 from the untreated control group and 21 from treated animals that yielded data at all three time points. All 15 control animals were sampled at each time point, so the sequencing table contains 14 control animals rather than 15; the sequences of one control animal are missing (see the note below). After removal of *Austrostrongylus victoriensis* and retention of the three target genera, the table contained 788 OTUs and was rarefied to 2120 reads per sample. Reads were assigned to *Trichostrongylus* (98.98% of retained reads on average; 132,199 reads in total), *Parascaris* (1359 reads) and *Coronocyclus* (two reads) (Supplementary Tables S2 and S6). No sequence assigned to any cyathostomin genus was recovered, and no *Strongylus* sequence was detected by 18S r2b amplicon sequencing.

**Figure 4:**
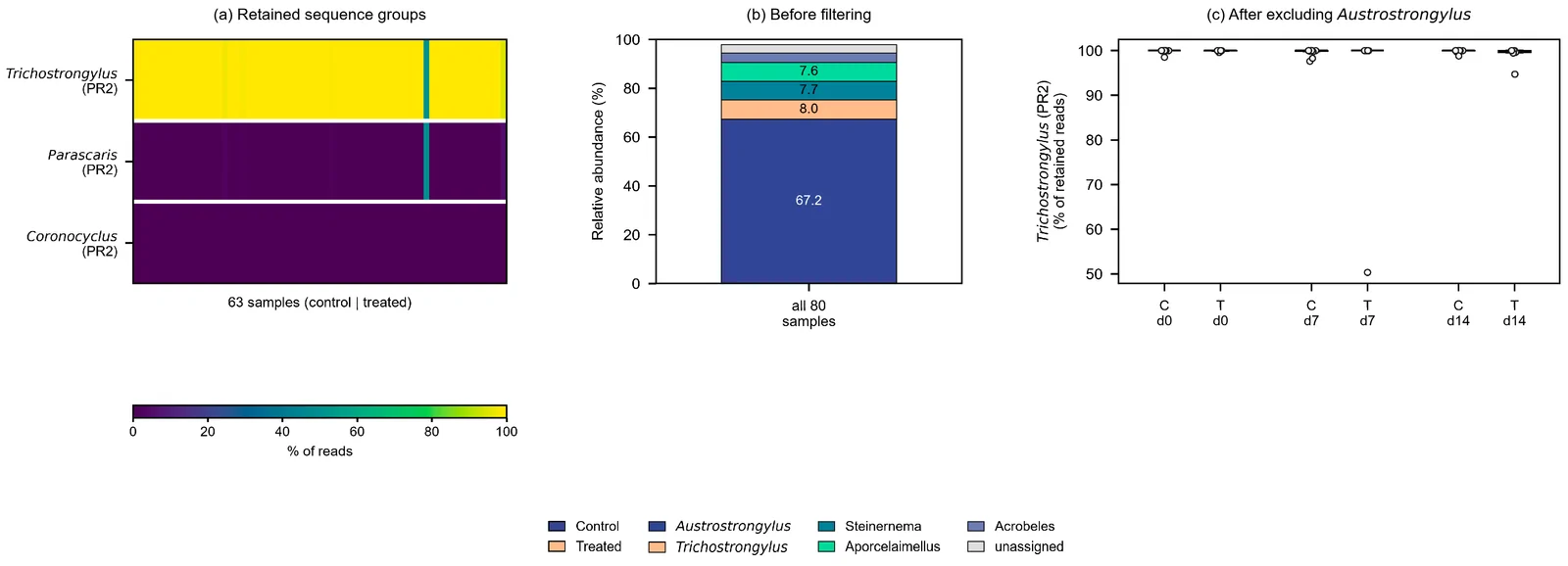
Community composition and the effect of the taxonomy filter. Genus names are PR2 assignments that the BLAST verification did not confirm (Section 3.4). (**a**) Relative abundance of the three retained sequence groups across the 63 sequenced samples, ordered by group and time point. (**b**) Mean composition of the complete, unfiltered community (80 samples) before removal of *Austrostrongylus victoriensis*. (**c**) Share of the *Trichostrongylus*-assigned group after that exclusion, by group and sampling day (C, control; T, treated); boxplots show the median and interquartile range with individual samples overlaid. Panels (**a**,**b**) are compared to the tests in Table S7.

Because the filter that produced this table removes a large part of the original community, the composition of the unfiltered data is important context. In the complete community table from the same bioinformatic run, covering 80 samples and 9397 OTUs rarefied to 62,383 reads per sample, *Austrostrongylus* accounted for 67.24% of all reads and was present in 80 of 80 samples; *Trichostrongylus* accounted for 7.95%, and a further ~20% of reads were assigned to free-living or soil-associated nematode genera such as *Steinernema*, *Aporcelaimellus*, *Acrobeles* and *Meloidogyne* (Supplementary Table S4; Figure 4). *Trichostrongylus* therefore becomes the group with the highest share of reads only after *Austrostrongylus* is removed; in the unfiltered table, *Austrostrongylus* holds 67.24% of reads and *Trichostrongylus* holds 7.95%.

The BLAST check qualifies these assignments. Of the 788 representative sequences, 787 returned a hit at 1 × 10^−20^. The best hits did not fall predominantly in *Trichostrongylus*, the genus assigned to 746 of the 788 OTUs: 729 matched *Spiculopteragia* most closely; 41 matched *Parascaris* and only three matched *Trichostrongylus*. More importantly, the leading genus was not separated from the runner-up: the median bitscore gap was 0.0, and for 751 of the 787 OTUs, carrying 132,218 of the 133,560 reads, the two leading genera were within 10 bits. Best-hit identity was 96.9% at the median and reached 100% for only 11 OTUs. The same sequences therefore receive different genus labels depending on the reference collection: PR2 returns *Trichostrongylus* whereas GenBank returns *Spiculopteragia* or *Cooperia*. The genus-level assignments are therefore reported as provisional.

### 3.5. Alpha Diversity

**Figure 5:**
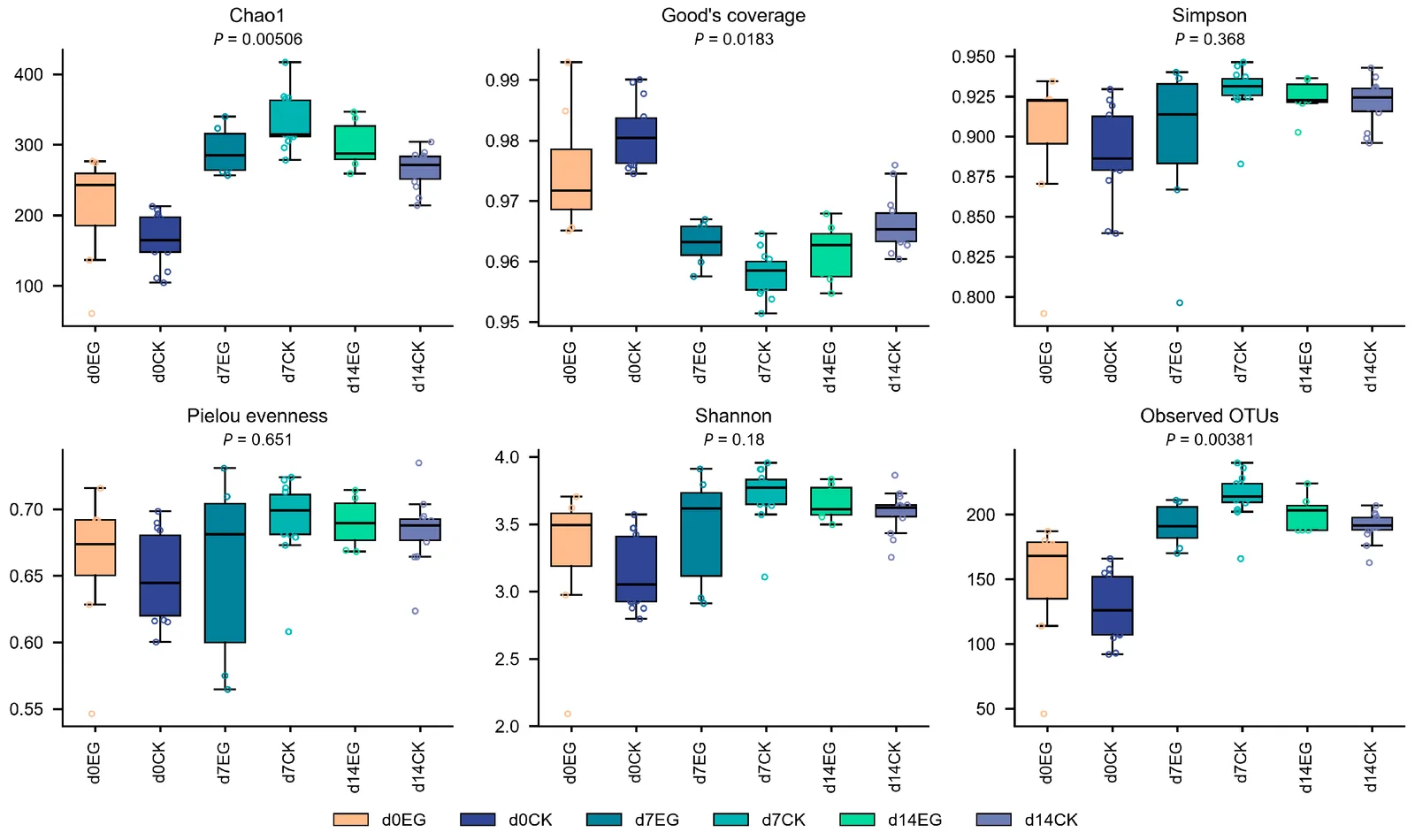
Alpha diversity of the nematode community recovered from eggs, computed on the table rarefied to 2120 reads per sample (*n* = 63 sequenced samples: 42 from the control group and 21 from treated animals). Panels show observed OTUs, Chao1, Shannon, Simpson, Pielou evenness and Good’s coverage; boxes show the median and interquartile range with individual samples overlaid. *p* values are from a linear mixed model (REML, statsmodels) of the form index ~ group × day with animal as a random intercept, with the interaction being tested by a Wald test. The group × day interaction is not significant for any index, whereas the day effect is significant for all diversity indices, indicating that the temporal increase in diversity occurred in the untreated control group as well as in the treated group. Good’s coverage is a completeness check rather than a diversity index and was not modelled.

Good’s coverage exceeded 0.95 in all samples (minimum 0.943), so the rarefied libraries describe the retained communities adequately (Figure 5). Alpha diversity increased over time in both groups: the day term of the mixed model was significant for observed OTUs (*p* = 3.36 × 10^−7^), Chao1 (*p* = 2.26 × 10^−5^), Shannon (*p* = 3.57 × 10^−5^), Simpson (*p* = 0.0017) and Pielou evenness (*p* = 0.0049). The group × day interaction was not significant for any index (*p* = 0.68–0.87) and neither was the group main effect (*p* = 0.52–0.97). Within-group *Friedman* tests confirmed the change in the untreated control animals as well as the treated animals (control χ^2^ = 26.14, *p* < 0.0001, Kendall’s W = 0.934; treated χ^2^ = 11.14, *p* = 0.0038). Diversity therefore rose in both groups and cannot be attributed to albendazole.

### 3.6. Beta Diversity

**Figure 6:**
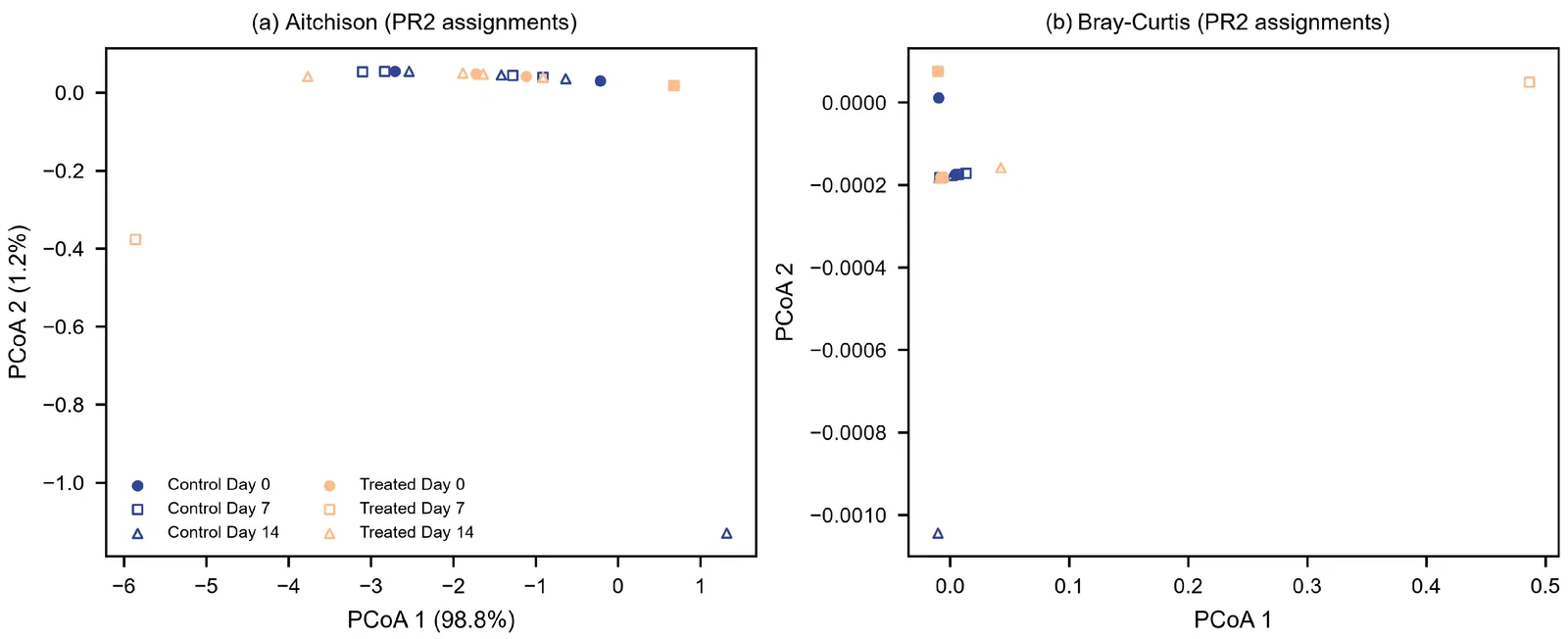
Principal coordinate analysis of genus-level nematode community composition (*n* = 63 sequenced samples) based on (**a**) Aitchison and (**b**) Bray–Curtis distances computed from the PR2-assigned sequence groups, whose genus labels the BLAST verification did not confirm (Section 3.4). Filled symbols denote untreated control samples and open symbols denote treated samples; colours denote sampling day. PERMANOVA was performed with 9999 permutations, restricted within animal for the day term and unrestricted for the group term; *PERMDISP* tested the homogeneity of multivariate dispersion.

Principal coordinates analysis of genus-level composition explained 99.0% of the variance on the first axis for the Aitchison distance (Figure 6). No effect of treatment group was found (PERMANOVA, R^2^ = 0.0332, *F* = 2.096, *p* = 0.159 for Aitchison; R^2^ = 0.0363, *F* = 2.298, *p* = 0.089 for Bray–Curtis), nor of sampling day with permutations restricted within animal (R^2^ = 0.0212, *F* = 0.650, *p* = 0.541; R^2^ = 0.0317, *F* = 0.981, *p* = 0.468), and the group × day term was not significant (*p* = 0.709 and *p* = 0.937). *PERMDISP* showed that dispersion differed between groups (Aitchison *F* = 5.382, *p* = 0.0192; Bray–Curtis *F* = 8.889, *p* = 0.0002), so the absence of a *PERMANOVA* group effect is an absence of a difference in centroid location rather than evidence that the groups are identically dispersed.

### 3.7. Genus-Level Abundance and Longitudinal Dynamics

Across the 63 samples, *Trichostrongylus* accounted for 98.98% of retained reads and was present in all 63 samples; *Parascaris* accounted for 1.02% and was present in 16 samples; *Coronocyclus* accounted for 0.00150% and was present in a single sample (Supplementary Table S6). In the *CLR* mixed model, no genus showed a significant group effect, day effect or group × day interaction after Benjamini–Hochberg correction: for *Trichostrongylus* the uncorrected *p* values were 0.767 (group), 0.637 (day) and 0.449 (interaction); for *Parascaris*, they were 0.833, 0.797 and 0.361; and for *Coronocyclus*, they were 0.910, 0.983 and 0.292. The *Friedman* companion test within the seven treated animals gave *p* = 0.204 for *Parascaris* and *p* = 0.204 for *Trichostrongylus*.

**Figure 7:**
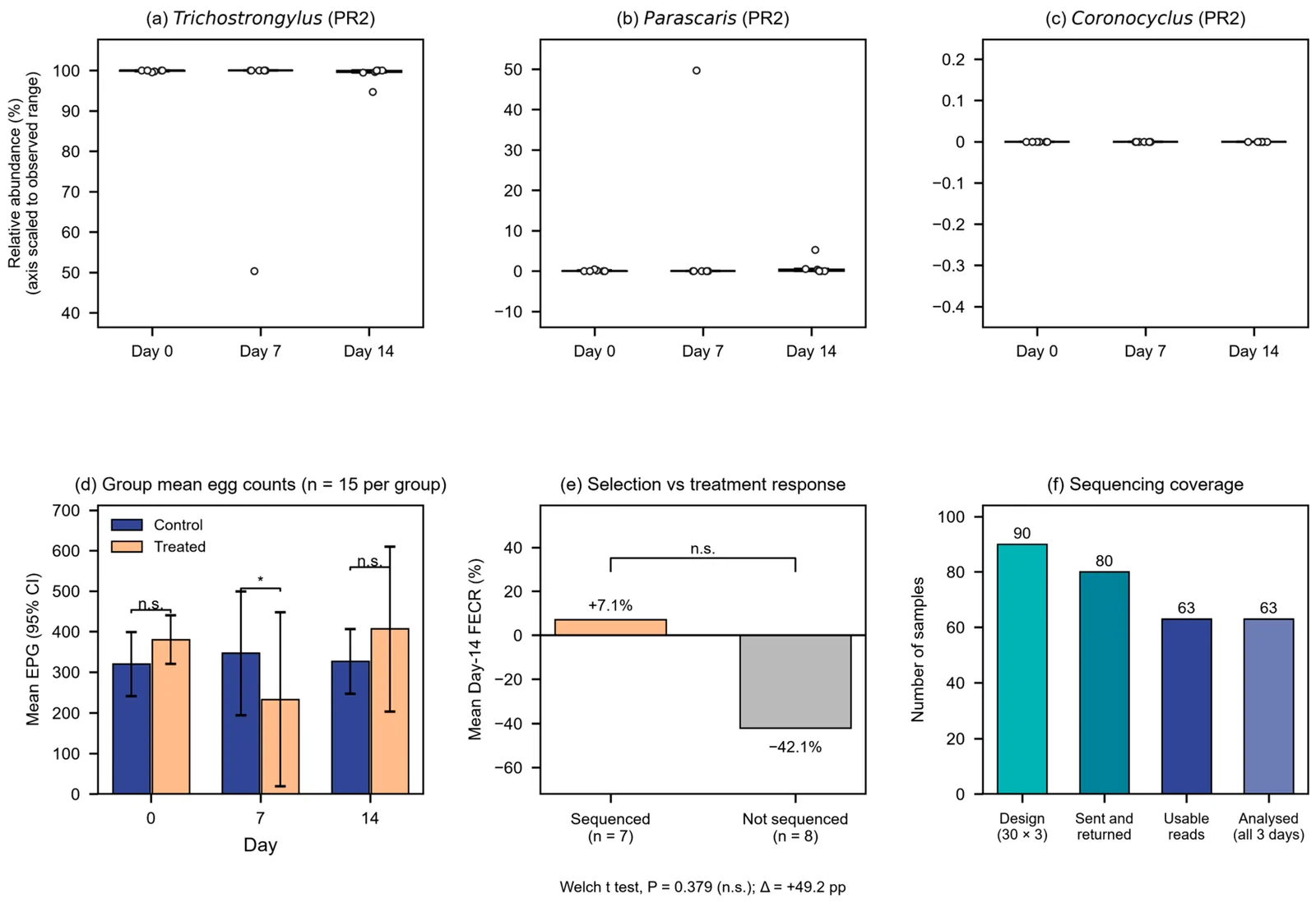
Genus-level sequence groups, group mean egg counts and sequencing coverage. Genus names are PR2 assignments that the BLAST verification did not confirm (Section 3.4). (**a**–**c**) Relative abundance of the three retained sequence groups in the treated animals, each on an axis scaled to the range observed for that group. (**d**) Group mean egg counts with 95% confidence intervals; brackets give *Mann*–*Whitney* comparisons at each day. (**e**) Mean Day 14 FECR of the seven treated animals with sequence data at all three time points versus the eight without (*Welch* *t* test, *p* = 0.379, *n.s.*). (**f**) Samples at each stage of the design. Significance convention as in Figure 2. Full statistics are in Tables S3, S6 and S7.

Two features of the genus-level data need explicit statement (Figure 7). First, *Parascaris* abundance is driven almost entirely by one sample: of 1359 *Parascaris* reads in the dataset, 1053 occur in sample d7EG1, where they are 49.7% of that sample’s reads. The apparent increase in mean *Parascaris* abundance on Day 7 (7.10% versus 0.09% on Day 0) rests on this single sample, and the *CLR* mixed model returned *p* = 0.797 for the day term in this group. Second, *Coronocyclus* is represented by two reads in one control sample; we report the detection without either discounting it on grounds of abundance or treating it as biologically meaningful, because a signal of this magnitude supports neither reading. No inter-genus replacement was evident, but with three genera and seven treated animals, the tests have low power.

### 3.8. Sequencing Coverage of the Treatment Group and Selection Bias

Of the 15 treated animals, only seven (numbers 1, 2, 3, 5, 6, 7 and 8) yielded sequence data at all three time points. Their Day 14 FECR values were 50%, −75%, 25%, 100%, 50%, 0% and −100%, so the subset spans the full range of responses rather than only the poor responders, but it is not a random sample. Seven of the 15 control animals are not represented either, because three control samples were lost to low read numbers. The molecular results therefore describe the sequenced subset and are reported as exploratory.

## 4. Discussion

### 4.1. Reduced Albendazole Efficacy in This Herd

The principal finding of this study is that albendazole did not reduce strongyle-type egg output in this herd. The Day 14 group-level FECR was −24.49%, the paired FECR was −7.02%, and the upper 90% confidence limit (19.93%) lay far below the 99% target efficacy that the 2023 WAAVP guideline specifies for benzimidazoles against cyathostomins in equids [28]. Under the guideline’s classification, this constitutes resistance of the nematode population on this farm. The result is consistent with reports of BZ resistance in equine and donkey strongyles from Norway [13], France [14], Ethiopia [15] and Romania [16], and it contrasts with the susceptible situation reported for Kermanshah Province, Iran [17].

Two qualifications are important. First, the study did not meet the guideline’s egg count precision requirement, as set out in Section 4.6: the direction of the classification is unlikely to be affected, because the point estimate is close to zero reduction and the confidence limit lies far below the threshold, but the magnitude should not be over-interpreted. Second, low efficacy in a single field trial does not by itself establish a genetic mechanism. Inadequate dosing, tablet administration and the pharmacokinetics of benzimidazoles in donkeys, which differ from those in horses [36], are plausible contributors that the design cannot separate. A further possibility, specific to donkeys, is that benzimidazoles are eliminated faster in this host than in horses [37], which would shorten the period of effective exposure. Because benzimidazole resistance in cyathostomins is associated with polymorphisms at codons 167 and 200 of β-tubulin isotype 1 [38], and because resistant populations can persist for many years [39], the finding has implications beyond this herd.

Because both the treated group and the untreated control group were included in the longitudinal models, the study can also state what it did not find: no group × time interaction was detectable in egg counts (*p* = 0.872), and no within-group change over time was detected in either group (*Friedman p* = 0.116 for treated and *p* = 0.646 for control animals). Egg counts of treated and untreated animals therefore followed statistically indistinguishable trajectories over the 14 days. This is the expected pattern when efficacy is close to zero, and it reinforces the phenotypic conclusion, but it means that no effect of treatment can be claimed at the level of individual animals.

Power and precision. Because the guideline classifies by the confidence limits rather than by a test against zero, the relevant operating characteristic is the probability of a correct classification. At the observed coefficients of variation, the standard error of the log ratio is 0.259, so the study declares resistance with 80% power for any true reduction at or below 98.8% and with 90% power at or below 98.9%. The observed −24.49% lies far below both, so the probability of misclassifying this herd is negligible. The converse is the substantive limitation: a 90% confidence interval spanning more than 113 percentage points means the same design could not have certified a genuinely effective product as susceptible. A group size of 15, which meets the guideline, is therefore adequate to detect failure of treatment but not to certify efficacy.

### 4.2. Egg Counts, Rebound and the Interpretation of Day-14 Values

Egg output was temporarily suppressed on Day 7 (group mean 233.3 EPG versus 380.0 on Day 0) before counts returned to their pre-treatment level by Day 14. Several non-exclusive explanations are compatible with the data: resumption of shedding by surviving parasites once drug pressure subsided, maturation of stages already present, natural fluctuation in faecal egg counts, counting error inherent in the McMaster method, and insufficient pharmacological exposure related to the pharmacokinetics of benzimidazoles in donkeys [36]. The design cannot distinguish among these, and the interpretation follows the established reading of FECRT data, in which elevated post-treatment counts are attributed to surviving parasites rather than to new infection within the observation window [40]. Attributing the increase to the proliferation of adult worms, or to reinfection becoming patent within the trial, is not supportable: adult nematodes do not proliferate in the intestinal lumen, and the pre-patent period of equine gastrointestinal nematodes is about six weeks.

### 4.3. The Sequencing Data Are Limited by Marker and by Sample Selection

The molecular component must be read with two constraints in view. The first is the marker. The 18S r2b region resolves equine strongylids poorly compared to ITS-2 [12], and every retained non-*Trichostrongylus* read was assigned to *Parascaris* or *Coronocyclus*: no cyathostomin genus was recovered and no *Strongylus* sequence was detected, although strongyle-type eggs were present in 62.16% of the screened animals. That more likely reflects primer bias and incomplete reference databases than a genuine absence of cyathostomins. The BLAST verification in Section 3.4 removes any remaining support for a biological reading: the classifier assigned 746 of 788 OTUs to *Trichostrongylus*, yet 729 matched *Spiculopteragia* most closely, and for 95% of the OTUs, carrying 99% of the reads, the leading genus was within 10 bits of the runner-up. *Spiculopteragia* parasitises cervids and *Trichostrongylus* mainly ruminants, so neither is plausible for a donkey. Genus-level composition is therefore not treated as established, and no conclusion rests on it. Species-level identification is not possible with this marker.

The second constraint is sample selection. Only seven of the 15 treated animals gave data at all three time points, and these were not a random subset: their mean day 14 FECR was +7.14%, against −42.08%, a difference of +49.2 percentage points in the eight that were not sequenced. The absence of a statistically significant difference between these subgroups does not establish equivalence, because the confidence interval of the difference is wide. Whatever the mechanism, the missing samples are not missing at random, and the sequencing results describe the seven sequenced animals only. The absence of significant differences in the community comparisons (all *p* > 0.15) must likewise not be read as evidence that the community was unchanged, because with three genera and seven treated animals, the tests have low power.

The community composition before and after the taxonomy filter adds a further caution. In the complete table from the same run, *Austrostrongylus victoriensis* accounted for 67.24% of reads across all 80 samples and *Trichostrongylus* for 7.95%, and about 20% of reads were assigned to free-living or soil-associated genera. The strongylid community of equids is normally dominated by cyathostomins with *Strongylus vulgaris* at lower frequency [41], so a profile in which one non-cyathostomin genus holds two thirds of the reads is not what this host would predict. *Austrostrongylus victoriensis* parasitises Australian macropods and its dominance here is not biologically plausible; misassignment or contamination are the likely explanations. This remains unresolved, which is one reason the molecular results are exploratory, and it means the dominance of *Trichostrongylus* is a property of the filtered table rather than of the original community.

Finally, the detection limit of the approach should be stated plainly. Amplicon sequencing of DNA extracted from concentrated eggs measures the relative abundance of egg-excreting taxa; it does not measure worm burden, and it cannot detect stages that are not shedding eggs. The two genera represented by essentially a single sample, *Parascaris* (one sample contributing 77% of its reads) and *Coronocyclus* (two reads in one sample), illustrate how easily a low-abundance taxon can generate a misleading pattern in a dataset of this size.

### 4.4. Microscopy and Sequencing as Complementary Methods

The strongyle-type eggs seen microscopically were not identified as *Strongylus*, and the absence of *Strongylus* sequences therefore does not contradict the microscopical findings. Equine strongylid eggs cannot be differentiated to genus by light microscopy [9], and the McMaster method and 18S r2b sequencing address different questions at different resolutions: the first establishes whether eggs are being shed and in what quantity; the second establishes which taxa contributed the DNA that amplified. They are complementary techniques, not mutual validations, and the concordance between them should not be assessed by comparing a morphotype count with a genus list.

### 4.5. Diversity Patterns Cannot Be Attributed to Treatment

Alpha diversity increased over the sampling period, but the increase was not specific to treated animals: the group × day interaction was not significant for any index (*p* = 0.68–0.87) and the same increase occurred in the untreated control group (Friedman *p* < 0.0001 for observed OTUs and Chao1, Kendall’s W = 0.93). Within-animal variation in the control group exceeded between-animal variation (mean *SD* 45.8 versus 18.1 observed OTUs). The pattern is therefore better explained by factors affecting all samples over time, including the low and declining biomass of egg-derived DNA, stochastic amplification and variation in sequencing depth, than by an ecological disturbance caused by albendazole. Explanations invoking the release of DNA from dead worms or release of rare taxa from competition are speculative and unsupported by the data. Parasite-community replacement has been proposed as a management tool for resistant nematode populations in other hosts [42], but nothing in the present dataset speaks to that possibility.

### 4.6. Limitations

The limitations are substantial. (1) The FECRT meets the guideline’s group-size requirement of 15 animals per group but not its egg count precision requirement: Day 0 counts total 57 eggs at a factor of 100 or 114 at a factor of 50, below the required mean of eight per animal, so the magnitude of the reduction is imprecise. (2) Only albendazole was tested; susceptibility to macrocyclic lactones and pyrantel is unknown [6,8,43]. (3) No β-tubulin genotyping was performed, so no genetic basis is claimed and a pharmacokinetic explanation cannot be excluded. (4) The 18S r2b marker resolves equine strongylids poorly; no cyathostomin genus was recovered and no *Strongylus* sequence was detected, so the results do not describe the farm’s nematode fauna. The BLAST verification in Section 3.4 shows the consequence: the leading genus was within 10 bits of the runner-up for 95% of the OTUs and the label changed with the reference collection, so the genus assignments are provisional and the apparent dominance of *Trichostrongylus* is an artefact of the reference database. (5) Only seven of 15 treated animals gave sequence data at all three time points and they responded differently from those that did not, so the molecular results are exploratory and apply to that subset. (6) Material was sent for sequencing only where the egg count was non-zero, with a zero count being taken to mean that no eggs were present. This was not a random selection: it concentrates the molecular analysis on animals shedding eggs continuously, excludes those that responded best, and means the molecular data cannot describe the treated group as a whole. A zero count may also reflect low or intermittent output rather than true absence. We regard this rule, and the resulting absence of egg-negative samples, as the principal weakness alongside the precision of the egg counts. (7) The unfiltered data are dominated by *Austrostrongylus victoriensis*, a sequence implausible for this host and locality. (8) The trial covered 14 days on one farm, so nothing can be concluded about other farms, seasons or the longer-term trajectory of resistance, and refugia-based strategies [44] cannot be evaluated from these data. The diversity and composition analyses also rest on three sequence groups and seven treated animals, so their power is low.

## 5. Conclusions

Albendazole showed very low efficacy against strongyle-type nematodes in these donkeys. The Day 14 FECR was −24.49% (90% *CI* −93.56 to 19.93), so egg output after treatment exceeded that of the untreated control group, and the upper confidence limit lay far below the 99% target efficacy for benzimidazoles in equids. Under Box 5 of the guideline, the population is therefore classified as resistant. The same conclusion follows from the paired estimate (−7.02%) and from the individual responses, and it does not depend on the multiplication factor used to convert chamber counts to EPG, because that factor cancels in the ratio.

## Figures and Tables

**Table 1:** Allocation of the 30 animals, body weight, dose administered and egg counts.

Animal	Group	Age (y)	Weight (kg)	Day 0 EPG	Day 7 EPG	Day 14 EPG	Albendazole (mg)	Tablets (50 mg)
1	Treated	1	100	200	200	100	1000	20
2	Treated	1	120	400	0	700	1200	24
3	Treated	1	110	400	100	300	1100	22
4	Treated	1	130	400	0	0	1300	26
5	Treated	1	100	600	0	0	1000	20
6	Treated	1	120	400	100	200	1200	24
7	Treated	1	110	500	500	500	1100	22
8	Treated	1	130	300	0	600	1300	26
9	Treated	1.5	100	300	0	400	1000	20
10	Treated	1.5	120	200	900	400	1200	24
11	Treated	1.5	110	500	0	100	1100	22
12	Treated	1.5	130	400	0	1200	1300	26
13	Treated	1.5	100	400	100	0	1000	20
14	Treated	1.5	120	300	300	1000	1200	24
15	Treated	1.5	110	400	1300	600	1100	22
16	Control	1	130	400	300	300	1300	26
17	Control	1	100	400	500	100	1000	20
18	Control	1	120	200	200	300	1200	24
19	Control	1	110	500	100	600	1100	22
20	Control	1	130	300	500	200	1300	26
21	Control	1	100	400	300	500	1000	20
22	Control	1	120	700	200	300	1200	24
23	Control	1	110	300	1100	300	1100	22
24	Control	1.5	130	300	200	500	1300	26
25	Control	1.5	100	200	500	200	1000	20
26	Control	1.5	120	300	700	500	1200	24
27	Control	1.5	110	200	200	200	1100	22
28	Control	1.5	130	200	100	400	1300	26
29	Control	1.5	100	200	100	300	1000	20
30	Control	1.5	120	200	200	200	1200	24

**Table 2:** Prevalence of gastrointestinal nematode egg morphotypes in 74 female donkeys on a single farm.

Egg Morphotype	Positive Samples	Infection Rate (%)	95% CI, Wilson (%)	95% CI, Clopper–Pearson (%)	EPG Range
Strongyle-type eggs	45 single + 1 mixed	62.16 (46/74)	50.77–72.35	50.13–73.19	100–1500
*Parascaris* spp. eggs	1 single + 1 mixed	2.70 (2/74)	0.74–9.33	0.33–9.42	100
Mixed infection (both morphotypes)	1	1.35 (1/74)	0.24–7.27	0.03–7.30	400 (combined total)
Any nematode egg (total positives)	47 (each counted once)	63.51 (47/74)	52.13–73.56	51.51–74.40	100–1500

Note: Each morphotype was counted independently, so a mixed-infection sample contributes to both morphotype categories while the total positive count counts it once; the morphotype counts (46 + 2) therefore sum to more than the 47 positive samples. The EPG of 400 for the mixed-infection sample is the combined total of both morphotypes. Confidence intervals were computed by the Wilson score method (primary) and the Clopper–Pearson exact method (for comparison). Prevalence refers to these 74 female donkeys on one farm and is not extrapolated to Xinjiang.

**Table 3:** Faecal egg count reduction and classification of anthelmintic resistance.

Group	*n*	Day 0 EPG (Mean ± *SD*)	Day 7 EPG (Mean)	Day 14 EPG (Mean ± *SD*)	FECR (%)	90% *CI* (%)	Classification
Albendazole-treated	15	380.0 ± 108.2	233.3	406.7 ± 367.4	−24.49	−93.56 to 19.93	Resistant
Untreated control	15	320.0 ± 142.4	346.7	326.7 ± 143.8	—	—	—

Note: FECR was calculated by the unpaired formula (Equation (4)) from arithmetic group means, and confidence limits by the log-scale method of Equations (5)–(7). The paired FECR on the same animals was −7.02%. Classification follows Box 5 of the 2023 WAAVP guideline [28]: target efficacy 99%, research-protocol lower efficacy target 95% (grey zone 95–99%), classification by the 90% confidence interval, with resistance declared when the upper 90% confidence limit falls below 99%. EPG values are those of the submitted dataset.

## Data Availability

The original contributions presented in this study are included in the article/supplementary material. Further inquiries can be directed to the corresponding author(s).

## Supplementary Materials

The following supporting information can be downloaded at https://www.mdpi.com/article/10.3390/vetsci13101104/s1, Figure S1. Rarefaction curves. Table S1. Individual egg counts and faecal egg count reduction rates for all 30 enrolled animals. Table S2. Per-sample read counts and observed OTUs for the 63 sequenced samples. Table S3. Comparison of the treated animals that yielded sequence data at all three time points with those that did not. Table S4. Complete taxonomic profile before and after filtering. Table S5. Alpha diversity indices for all 63 samples. Table S6. Genus-level relative abundance by group and time point with *CLR* model results. Table S7. *PERMANOVA* and *PERMDISP* results. Table S8. Status of laboratory and procedural metadata. Table S9. BLAST verification of the 788 representative OTU sequences. Table S10. Per-sample laboratory metadata for all 30 enrolled animals. Table S11. Animal allocation, body weight and dose administered.
