# Supplementary material for: Benzimidazole Resistance of Donkey Gastrointestinal Nematodes in Xinjiang, China, Assessed by the Faecal Egg Count Reduction Test and 18S r2b Amplicon Sequencing
Source: Vet Sci. 2026 Oct 16;13(10):1104. doi: 10.3390/vetsci13101104 (PMC13709559; doi:10.3390/vetsci13101104)
Supplement: Supplementary file 1 [file vetsci-13-01104-s001.zip › vetsci-4593314-supplementary.pdf]

Supplementary Materials

Benzimidazole Resistance of Donkey Gastrointestinal Nematodes in Xinjiang, China, Assessed by the Faecal Egg Count Reduction Test and 18S r2b Amplicon Sequencing

This file contains Figure S1 and Tables S1–S9. Tables S1, S2, S5, S6 and S9 are also supplied in machine-readable form in the accompanying workbook and as individual files.

Figure S1. Rarefaction curves

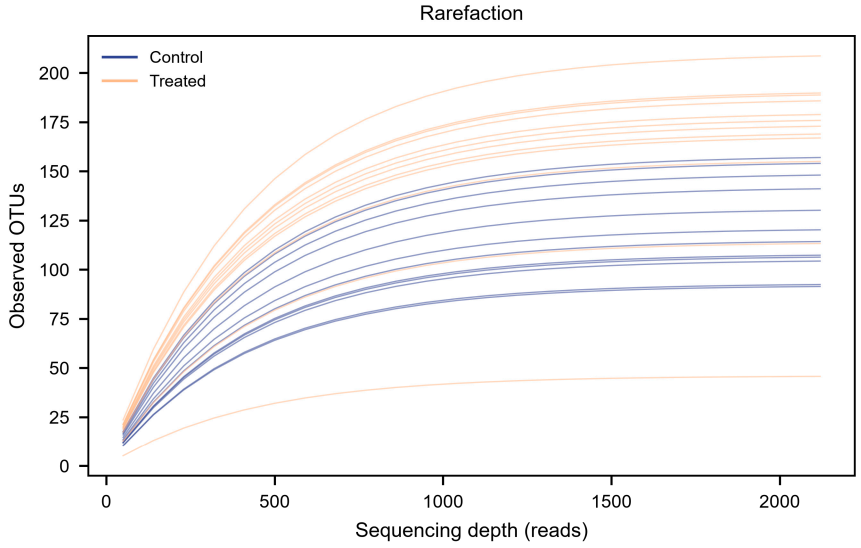

Figure S1. Rarefaction curves for (a) the primary table used for all diversity analyses (63 samples, 788 OTUs after removal of *Austrostrongylus victoriensis* and restriction to three genera, rarefied to 2,120 reads per sample) and (b) the complete community table from the same bioinformatic run (80 samples, 9,397 OTUs, rarefied to 62,383 reads per sample). Each line is one sample; blue, untreated control; red, albendazole-treated. Curves approach an asymptote within the sequenced depth, indicating that the retained communities were sampled adequately, which is corroborated by Good's coverage above 0.95 in every sample (Table S5). Curves were computed by resampling reads without replacement (NumPy, seed 42).

Table S1. Individual faecal egg counts and faecal egg count reduction rates for all 30 enrolled animals.

Animal No.	Group	Day 0 EPG	Day 7 EPG	Day 14 EPG	Day 7 FECR (%)	Day 14 FECR (%)
1	Treated	200	200	100	0.0	50.0
2	Treated	400	0	700	100.0	−75.0
3	Treated	400	100	300	75.0	25.0
4	Treated	400	0	0	100.0	100.0
5	Treated	600	0	0	100.0	100.0
6	Treated	400	100	200	75.0	50.0
7	Treated	500	500	500	0.0	0.0
8	Treated	300	0	600	100.0	−100.0
9	Treated	300	0	400	100.0	−33.3
10	Treated	200	900	400	−350.0	−100.0
11	Treated	500	0	100	100.0	80.0
12	Treated	400	0	1200	100.0	−200.0
13	Treated	400	100	0	75.0	100.0
14	Treated	300	300	1000	0.0	−233.3
15	Treated	400	1300	600	−225.0	−50.0
16	Control	400	300	300	25.0	25.0
17	Control	400	500	100	−25.0	75.0
18	Control	200	200	300	0.0	−50.0
19	Control	500	100	600	80.0	−20.0
20	Control	300	500	200	−66.7	33.3
21	Control	400	300	500	25.0	−25.0
22	Control	700	200	300	71.4	57.1

23	Control	300	1100	300	−266.7	0.0
24	Control	300	200	500	33.3	−66.7
25	Control	200	500	200	−150.0	0.0
26	Control	300	700	500	−133.3	−66.7
27	Control	200	200	200	0.0	0.0
28	Control	200	100	400	50.0	−100.0
29	Control	200	100	300	50.0	−50.0
30	Control	200	200	200	0.0	0.0

Note: *EPG = (eggs in the left chamber + eggs in the right chamber) × 100. Each sample was counted once by a single operator blinded to group allocation, so the tabulated value is the count from that one examination. Individual FECR values are descriptive only; group efficacy is estimated by the group-level FECR in Table 2. The Day-7 EPG of animal 7 was adjudicated as 500 and this value is used throughout; a provisional entry of 3800 for the same animal in an intermediate laboratory workbook was superseded.*

Table S2. Per-sample read counts and observed OTUs for the 63 sequenced samples (rarefied to 2,120 reads per sample).

Sample	Group	Day	Animal	Total reads	*Trichostrongylus*	*Parascaris*	*Coronocyclus*	Observed OTUs
d0CK1	Control	0	16	2120	2089	31	0	92
d0CK2	Control	0	17	2120	2120	0	0	93
d0CK3	Control	0	18	2120	2120	0	0	108
d0CK4	Control	0	19	2120	2120	0	0	105
d0CK5	Control	0	20	2120	2120	0	0	115
d0CK6	Control	0	21	2120	2120	0	0	121
d0CK7	Control	0	22	2120	2120	0	0	107
d0CK8	Control	0	23	2120	2120	0	0	131
d0CK10	Control	0	25	2120	2120	0	0	155
d0CK11	Control	0	26	2120	2119	1	0	149
d0CK12	Control	0	27	2120	2120	0	0	142
d0CK13	Control	0	28	2120	2120	0	0	158
d0CK14	Control	0	29	2120	2120	0	0	166
d0CK15	Control	0	30	2120	2120	0	0	153
d0EG1	Treated	0	1	2120	2116	4	0	46
d0EG2	Treated	0	2	2120	2120	0	0	114
d0EG3	Treated	0	3	2120	2120	0	0	156
d0EG5	Treated	0	5	2120	2120	0	0	168
d0EG6	Treated	0	6	2120	2111	9	0	177
d0EG7	Treated	0	7	2120	2120	0	0	187
d0EG8	Treated	0	8	2120	2120	0	0	180
d7CK1	Control	7	16	2120	2120	0	0	214
d7CK2	Control	7	17	2120	2120	0	0	218
d7CK3	Control	7	18	2120	2120	0	0	224
d7CK4	Control	7	19	2120	2120	0	0	214
d7CK5	Control	7	20	2120	2120	0	0	228
d7CK6	Control	7	21	2120	2120	0	0	166
d7CK7	Control	7	22	2120	2120	0	0	202
d7CK8	Control	7	23	2120	2120	0	0	224
d7CK10	Control	7	25	2120	2070	50	0	204
d7CK11	Control	7	26	2120	2117	3	0	211
d7CK12	Control	7	27	2120	2115	5	0	236
d7CK13	Control	7	28	2120	2084	36	0	240
d7CK14	Control	7	29	2120	2120	0	0	212
d7CK15	Control	7	30	2120	2120	0	0	209
d7EG1	Treated	7	1	2120	1067	1053	0	174
d7EG2	Treated	7	2	2120	2120	0	0	170
d7EG3	Treated	7	3	2120	2120	0	0	190
d7EG5	Treated	7	5	2120	2120	0	0	210
d7EG6	Treated	7	6	2120	2120	0	0	191
d7EG7	Treated	7	7	2120	2120	0	0	202
d7EG8	Treated	7	8	2120	2120	0	0	211
d14CK1	Control	14	16	2120	2120	0	0	192
d14CK2	Control	14	17	2120	2120	0	0	198
d14CK3	Control	14	18	2120	2120	0	0	176
d14CK4	Control	14	19	2120	2120	0	0	200
d14CK5	Control	14	20	2120	2120	0	0	185
d14CK6	Control	14	21	2120	2120	0	0	189
d14CK7	Control	14	22	2120	2118	0	2	188
d14CK8	Control	14	23	2120	2120	0	0	191
d14CK10	Control	14	25	2120	2118	2	0	190
d14CK11	Control	14	26	2120	2114	6	0	194

d14CK12	Control	14	27	2120	2120	0	0	201
d14CK13	Control	14	28	2120	2120	0	0	197
d14CK14	Control	14	29	2120	2095	25	0	207
d14CK15	Control	14	30	2120	2120	0	0	163
d14EG1	Treated	14	1	2120	2120	0	0	188
d14EG2	Treated	14	2	2120	2112	8	0	188
d14EG3	Treated	14	3	2120	2120	0	0	188
d14EG5	Treated	14	5	2120	2117	3	0	224
d14EG6	Treated	14	6	2120	2109	11	0	204
d14EG7	Treated	14	7	2120	2120	0	0	203
d14EG8	Treated	14	8	2120	2008	112	0	210

Note: *Every sample was rarefied to 2,120 reads. Samples from treated animals that do not appear at all three time points are absent because they did not yield sequence data at every time point; see Table S3. The sequencing table contains 14 control animals; the 15th control animal of the FECRT has no sequence data (see the manuscript). Genus names are PR2 assignments and are used as labels for sequence groups; the BLAST verification in Section 3.4 and Table S9 did not confirm them.*

Table S3. Comparison of treated animals that yielded sequence data at all three time points with those that did not.

Animal No.	Sequenced at all 3 time points	Day 0 EPG	Day 7 EPG	Day 14 EPG	Day 14 FECR (%)
1	Yes	200	200	100	50.00
2	Yes	400	0	700	−75.00
3	Yes	400	100	300	25.00
5	Yes	600	0	0	100.00
6	Yes	400	100	200	50.00
7	Yes	500	500	500	0.00
8	Yes	300	0	600	−100.00
4	No	400	0	0	100.00
9	No	300	0	400	−33.33
10	No	200	900	400	−100.00
11	No	500	0	100	80.00
12	No	400	0	1200	−200.00
13	No	400	100	0	100.00
14	No	300	300	1000	−233.33
15	No	400	1300	600	−50.00
Mean, sequenced (*n* = 7)	—	400.0	—	—	+7.14
Mean, not sequenced (*n* = 8)	—	362.5	—	—	−42.08

Note: *Baseline egg counts did not differ (Mann–Whitney U, P = 0.581), whereas the mean Day-14 FECR differed by +49.2 percentage points (95% CI −56.0 to 154.5; Welch t test P = 0.379; Mann–Whitney U P = 0.642). The difference is not statistically significant, but the interval is wide, so the sequenced subgroup cannot be assumed representative of the treatment group.*

Table S4. Complete taxonomic profile before and after filtering.

Genus	Reads (complete community, 80 samples)	% of complete community	Samples present	Retained after filtering	Reads (retained, 63 samples)	% of retained community
Austrostrongylus	3355470	67.2353	80/80	No	0	0.0000
Trichostrongylus	396884	7.9526	80/80	Yes	132199	98.9810
Steinernema	383040	7.6752	79/80	No	0	0.0000
Aporcelaimellus	381482	7.6439	80/80	No	0	0.0000
Acrobeles	199861	4.0047	80/80	No	0	0.0000
unassigned	170467	3.4157	80/80	No	0	0.0000
Stephanurus	22132	0.4435	79/80	No	0	0.0000
Meloidogyne	16005	0.3207	70/80	No	0	0.0000
Parascaris	14614	0.2928	28/80	Yes	1359	1.0175
Ancylostoma	11060	0.2216	77/80	No	0	0.0000
Hexamermis	9860	0.1976	76/80	No	0	0.0000
Alaimus	7809	0.1565	61/80	No	0	0.0000
Pristionchus	3268	0.0655	38/80	No	0	0.0000
Bathylaimus	2213	0.0443	50/80	No	0	0.0000
Poikilolaimus	2159	0.0433	24/80	No	0	0.0000
Anoplostoma	1983	0.0397	42/80	No	0	0.0000
Deletrocephalus	1478	0.0296	78/80	No	0	0.0000
Oscheius	1241	0.0249	79/80	No	0	0.0000
Rhabdolaimus	967	0.0194	39/80	No	0	0.0000
Neodiplogaster	933	0.0187	66/80	No	0	0.0000
Uncinaria	670	0.0134	66/80	No	0	0.0000
Myolaimus	646	0.0129	5/80	No	0	0.0000

Necator	619	0.0124	60/80	No	0	0.0000
Criconemoides	367	0.0074	16/80	No	0	0.0000
Oncholaimus	358	0.0072	9/80	No	0	0.0000
Epitobrilus	352	0.0071	9/80	No	0	0.0000
Acanthocheilonema	315	0.0063	8/80	No	0	0.0000
Litoditis	315	0.0063	63/80	No	0	0.0000
Paratrichodorus	240	0.0048	14/80	No	0	0.0000
Subanguina	200	0.0040	42/80	No	0	0.0000
Vexillata	191	0.0038	4/80	No	0	0.0000
Brugia	181	0.0036	18/80	No	0	0.0000
Litomosoides	167	0.0033	36/80	No	0	0.0000
Oxystomina	166	0.0033	15/80	No	0	0.0000
Rumenfilaria	158	0.0032	34/80	No	0	0.0000
Heterorhabditis	153	0.0031	32/80	No	0	0.0000
Cephaloboides	145	0.0029	19/80	No	0	0.0000
Fictor	134	0.0027	31/80	No	0	0.0000
Zoniolaimus	131	0.0026	43/80	No	0	0.0000
Cattiena	121	0.0024	21/80	No	0	0.0000
Hemicriconemoides	114	0.0023	43/80	No	0	0.0000
Tokorhabditis	105	0.0021	32/80	No	0	0.0000
Phanodermopsis	97	0.0019	24/80	No	0	0.0000
Heligmosomoides	89	0.0018	38/80	No	0	0.0000
Halalaimus	86	0.0017	6/80	No	0	0.0000
Monoposthia	79	0.0016	26/80	No	0	0.0000
Oswaldocruzia	78	0.0016	47/80	No	0	0.0000
Filarinema	76	0.0015	4/80	No	0	0.0000
Prodesmodora	75	0.0015	2/80	No	0	0.0000
Eurystomina	70	0.0014	21/80	No	0	0.0000
Crenosoma	60	0.0012	14/80	No	0	0.0000
Viscosia	58	0.0012	8/80	No	0	0.0000
Auanema	53	0.0011	13/80	No	0	0.0000
Sudhausia	51	0.0010	7/80	No	0	0.0000
Halomonhystera	51	0.0010	11/80	No	0	0.0000
Raphidascaroides	47	0.0009	25/80	No	0	0.0000
Gongylonema	44	0.0009	3/80	No	0	0.0000
Heterakis	38	0.0008	7/80	No	0	0.0000
Raillietnema	34	0.0007	9/80	No	0	0.0000
Pseudaphelenchus	33	0.0007	3/80	No	0	0.0000
Ostertagia	31	0.0006	15/80	No	0	0.0000
Globodera	29	0.0006	5/80	No	0	0.0000
Eutobrilus	28	0.0006	3/80	No	0	0.0000
Rhabditoides	23	0.0005	16/80	No	0	0.0000
Diplogaster	23	0.0005	3/80	No	0	0.0000
Hassalstrongylus	22	0.0004	13/80	No	0	0.0000
Diplogastrellus	22	0.0004	9/80	No	0	0.0000
Myctolaimus	22	0.0004	4/80	No	0	0.0000
Phasmarhabditis	21	0.0004	2/80	No	0	0.0000
Aspiculuris	20	0.0004	1/80	No	0	0.0000
Nematodirus	19	0.0004	3/80	No	0	0.0000
Parasitodiplogaster	19	0.0004	11/80	No	0	0.0000
Mesorhabditis	18	0.0004	14/80	No	0	0.0000
Haemonchus	17	0.0003	13/80	No	0	0.0000
Nothocriconemoides	16	0.0003	2/80	No	0	0.0000
Eubostrichus	16	0.0003	4/80	No	0	0.0000
Panagrolaimus	15	0.0003	2/80	No	0	0.0000
Cylindrolaimus	15	0.0003	2/80	No	0	0.0000
Parapristionchus	14	0.0003	1/80	No	0	0.0000
Ichtyobronema	14	0.0003	9/80	No	0	0.0000
Krefftascaris	14	0.0003	8/80	No	0	0.0000
Dorylaimellus	13	0.0003	11/80	No	0	0.0000
Caenorhabditis	13	0.0003	10/80	No	0	0.0000
Hovorkonema	13	0.0003	8/80	No	0	0.0000
Mammomonogamus	13	0.0003	9/80	No	0	0.0000
Teratorhabditis	12	0.0002	9/80	No	0	0.0000
Oigolaimella	10	0.0002	2/80	No	0	0.0000
Odilia	10	0.0002	4/80	No	0	0.0000
Tylencholaimus	10	0.0002	8/80	No	0	0.0000
Laxus	10	0.0002	4/80	No	0	0.0000
Hemicycliophora	9	0.0002	5/80	No	0	0.0000
Leptonchus	9	0.0002	7/80	No	0	0.0000

Grassenema	8	0.0002	2/80	No	0	0.0000
Heterodera	7	0.0001	4/80	No	0	0.0000
Metarhabditis	7	0.0001	2/80	No	0	0.0000
Ditylenchus	7	0.0001	2/80	No	0	0.0000
Micoletzkya	6	0.0001	3/80	No	0	0.0000
Xiphinema	6	0.0001	4/80	No	0	0.0000
Rhabditis	6	0.0001	1/80	No	0	0.0000
Ironus	6	0.0001	3/80	No	0	0.0000
Chylorhabditis	6	0.0001	1/80	No	0	0.0000
Acrobeloides	5	0.0001	5/80	No	0	0.0000
Funaria	5	0.0001	4/80	No	0	0.0000
Angiostrongylus	5	0.0001	4/80	No	0	0.0000
Amblydorylaimus	5	0.0001	5/80	No	0	0.0000
Chronogaster	5	0.0001	1/80	No	0	0.0000
Madathamugadia	5	0.0001	4/80	No	0	0.0000
Desmodora	5	0.0001	5/80	No	0	0.0000
Trichuris	4	0.0001	3/80	No	0	0.0000
Kalicephalus	4	0.0001	3/80	No	0	0.0000
Crustorhabditis	4	0.0001	4/80	No	0	0.0000
Rotylenchus	4	0.0001	3/80	No	0	0.0000
Thornenema	4	0.0001	2/80	No	0	0.0000
Mesodorylaimus	4	0.0001	3/80	No	0	0.0000
Toxascaris	4	0.0001	1/80	No	0	0.0000
Aglenchus	3	0.0001	1/80	No	0	0.0000
Phanoderma	3	0.0001	1/80	No	0	0.0000
Cephalenchus	3	0.0001	2/80	No	0	0.0000
Aphelenchoides	3	0.0001	1/80	No	0	0.0000
Aporcella	3	0.0001	1/80	No	0	0.0000
Brevibucca	3	0.0001	1/80	No	0	0.0000
Campydora	3	0.0001	3/80	No	0	0.0000
Filenchus	3	0.0001	2/80	No	0	0.0000
Ichthyocephaloides	3	0.0001	2/80	No	0	0.0000
Coslenchus	3	0.0001	1/80	No	0	0.0000
Teratocephalus	3	0.0001	3/80	No	0	0.0000
Orientatractis	3	0.0001	2/80	No	0	0.0000
Oesophagostomum	3	0.0001	1/80	No	0	0.0000
Pellioditis	3	0.0001	2/80	No	0	0.0000
Praeacanthonchus	3	0.0001	3/80	No	0	0.0000
Enoplus	3	0.0001	2/80	No	0	0.0000
Tripyloides	2	0.0000	1/80	No	0	0.0000
Bathyeurystomina	2	0.0000	1/80	No	0	0.0000
Ascaris	2	0.0000	2/80	No	0	0.0000
Diplogasteroides	2	0.0000	1/80	No	0	0.0000
Diplopeltis	2	0.0000	1/80	No	0	0.0000
Coronocyclus	2	0.0000	1/80	Yes	2	0.0015
Meyersia	2	0.0000	1/80	No	0	0.0000
Tylenchorhynchus	2	0.0000	2/80	No	0	0.0000
Prismatolaimus	2	0.0000	1/80	No	0	0.0000
Syringolaimus	2	0.0000	2/80	No	0	0.0000
Trichinella	2	0.0000	1/80	No	0	0.0000
Mononchoides	2	0.0000	1/80	No	0	0.0000
Discolaimus	2	0.0000	2/80	No	0	0.0000
Nippostrongylus	2	0.0000	1/80	No	0	0.0000
Aelurostrongylus	1	0.0000	1/80	No	0	0.0000
Mehdinema	1	0.0000	1/80	No	0	0.0000
Lenisaphelenchus	1	0.0000	1/80	No	0	0.0000
Hypodontus	1	0.0000	1/80	No	0	0.0000
Ceramonema	1	0.0000	1/80	No	0	0.0000
Discocriconemella	1	0.0000	1/80	No	0	0.0000
Odontolaimus	1	0.0000	1/80	No	0	0.0000
Syngamus	1	0.0000	1/80	No	0	0.0000
Rotylenchulus	1	0.0000	1/80	No	0	0.0000

Note: The complete-community columns come from table_f_even_depth_w_tax.xls of the 80-sample QIIME 2 run (9,397 OTUs, rarefied to 62,383 reads per sample). The retained columns come from the primary table (63 samples, 788 OTUs, rarefied to 2,120 reads per sample) after removal of *Austrostrongylus victoriensis*restriction to *TrichostrongylusParascaris* and *Coronocyclus*and removal of OTUs with fewer than 10 reads in total. *Austrostrongylus* accounts for the majority of the complete community and is present in all 80 samples; the grounds for its removal are discussed in the response to (, 19 and 25). Taxonomic labels are reproduced verbatim as returned by the PR2 database (v4.14.0); where a label includes a species epithet, for example the species-level assignment of one *Parascaris* OTU, this reflects the database entry and not a species-level identification by the present study. Throughout the manuscript the eggs and sequences are therefore reported at genus level

as *Parascaris spp.*consistent with (). Genus names are PR2 assignments and are used as labels for sequence groups; the BLAST verification in Section 3.4 and Table S9 did not confirm them.

Table S5. Alpha diversity indices for all 63 sequenced samples.

Sample	Group	Day	Anima l	Observed OTUs	Chao1	Shannon	Simpson	Pielou evenness	Good's coverage
d0CK1	Control	0	16	92	111.25	2.924	0.8727	0.6467	0.9896
d0CK2	Control	0	17	93	104.67	2.796	0.8396	0.6169	0.9901
d0CK3	Control	0	18	108	148.07	3.009	0.8860	0.6427	0.9840
d0CK4	Control	0	19	105	162.27	2.931	0.8799	0.6299	0.9830
d0CK5	Control	0	20	115	148.30	2.924	0.8825	0.6162	0.9825
d0CK6	Control	0	21	121	162.00	2.879	0.8409	0.6003	0.9802
d0CK7	Control	0	22	107	120.00	2.876	0.8790	0.6154	0.9877
d0CK8	Control	0	23	131	166.65	3.095	0.8864	0.6349	0.9807
d0CK10	Control	0	25	155	212.65	3.375	0.9085	0.6693	0.9755
d0CK11	Control	0	26	149	196.00	3.423	0.9193	0.6841	0.9774
d0CK12	Control	0	27	142	189.22	3.313	0.9100	0.6685	0.9759
d0CK13	Control	0	28	158	207.34	3.473	0.9228	0.6860	0.9745
d0CK14	Control	0	29	166	202.43	3.570	0.9295	0.6984	0.9759
d0CK15	Control	0	30	153	198.00	3.469	0.9135	0.6896	0.9788
d0EG1	Treated	0	1	46	61.00	2.091	0.7897	0.5463	0.9929
d0EG2	Treated	0	2	114	136.55	2.976	0.8704	0.6283	0.9849
d0EG3	Treated	0	3	156	244.50	3.402	0.9225	0.6737	0.9717
d0EG5	Treated	0	5	168	233.56	3.544	0.9233	0.6917	0.9717
d0EG6	Treated	0	6	177	242.81	3.704	0.9344	0.7157	0.9722
d0EG7	Treated	0	7	187	274.60	3.620	0.9226	0.6920	0.9656
d0EG8	Treated	0	8	180	276.46	3.493	0.9209	0.6726	0.9651
d7CK1	Control	7	16	214	278.53	3.842	0.9331	0.7160	0.9646
d7CK2	Control	7	17	218	313.70	3.782	0.9284	0.7024	0.9585
d7CK3	Control	7	18	224	295.65	3.907	0.9384	0.7220	0.9627
d7CK4	Control	7	19	214	314.41	3.654	0.9307	0.6809	0.9580
d7CK5	Control	7	20	228	334.61	3.804	0.9319	0.7007	0.9557
d7CK6	Control	7	21	166	366.18	3.109	0.8828	0.6081	0.9608
d7CK7	Control	7	22	202	314.59	3.572	0.9233	0.6728	0.9585
d7CK8	Control	7	23	224	305.33	3.774	0.9289	0.6973	0.9590
d7CK10	Control	7	25	204	416.62	3.682	0.9329	0.6924	0.9552
d7CK11	Control	7	26	211	368.24	3.647	0.9247	0.6815	0.9547
d7CK12	Control	7	27	236	313.94	3.956	0.9463	0.7240	0.9590
d7CK13	Control	7	28	240	365.07	3.908	0.9441	0.7130	0.9514
d7CK14	Control	7	29	212	356.03	3.637	0.9248	0.6790	0.9538
d7CK15	Control	7	30	209	311.53	3.767	0.9372	0.7052	0.9604
d7EG1	Treated	7	1	174	256.45	2.913	0.7963	0.5646	0.9660
d7EG2	Treated	7	2	170	340.00	2.952	0.8669	0.5749	0.9599
d7EG3	Treated	7	3	190	261.82	3.279	0.8998	0.6250	0.9623
d7EG5	Treated	7	5	210	285.07	3.794	0.9364	0.7096	0.9632
d7EG6	Treated	7	6	191	266.47	3.672	0.9138	0.6991	0.9670
d7EG7	Treated	7	7	202	323.36	3.615	0.9292	0.6811	0.9575
d7EG8	Treated	7	8	211	308.33	3.912	0.9401	0.7309	0.9656
d14CK1	Control	14	16	192	224.69	3.863	0.9427	0.7348	0.9759
d14CK2	Control	14	17	198	289.00	3.637	0.9182	0.6878	0.9632
d14CK3	Control	14	18	176	247.72	3.434	0.8990	0.6641	0.9693
d14CK4	Control	14	19	200	279.08	3.728	0.9371	0.7036	0.9637
d14CK5	Control	14	20	185	285.64	3.255	0.9020	0.6236	0.9613
d14CK6	Control	14	21	189	262.00	3.605	0.9251	0.6878	0.9656
d14CK7	Control	14	22	188	240.64	3.586	0.9180	0.6848	0.9684
d14CK8	Control	14	23	191	272.28	3.547	0.9149	0.6754	0.9637
d14CK10	Control	14	25	190	269.88	3.637	0.9304	0.6931	0.9660
d14CK11	Control	14	26	194	304.04	3.643	0.9258	0.6915	0.9627
d14CK12	Control	14	27	201	282.07	3.607	0.9295	0.6802	0.9604
d14CK13	Control	14	28	197	266.00	3.641	0.9233	0.6891	0.9670
d14CK14	Control	14	29	207	284.17	3.705	0.9309	0.6948	0.9651
d14CK15	Control	14	30	163	214.11	3.384	0.8960	0.6643	0.9745
d14EG1	Treated	14	1	188	285.53	3.584	0.9223	0.6845	0.9637
d14EG2	Treated	14	2	188	287.39	3.498	0.9227	0.6681	0.9627
d14EG3	Treated	14	3	188	259.19	3.611	0.9208	0.6895	0.9679
d14EG5	Treated	14	5	224	338.00	3.834	0.9365	0.7084	0.9547
d14EG6	Treated	14	6	204	273.16	3.799	0.9356	0.7143	0.9656
d14EG7	Treated	14	7	203	314.89	3.555	0.9026	0.6690	0.9580
d14EG8	Treated	14	8	210	346.50	3.745	0.9294	0.7005	0.9571

Note: Computed on the table rarefied to 2,120 reads per sample. Good's coverage exceeded 0.95 in all samples (minimum 0.943). 'Observed OTUs' replaces the term 'observed species' used in the previous version, in line with ().

Table S6. Genus-level relative abundance by group and time point, with centre-log-ratio mixed-model results.

Genus	Total reads	Prevalence	Control D0 mean %	Control D7 mean %	Control D14 mean %	Treated D0 mean %	Treated D7 mean %	Treated D14 mean %	Control D0 max %	Control D7 max %	Control D14 max %	Treated D0 max %	Treated D7 max %	Treated D14 max %	Group P	Day P	Group × day P	Group P (BH-FDR)	Day P (BH-FDR)	Group × day P (BH-FDR)
Coronocyclus	2	1/63	0.000	0.000	0.000	0.000	0.007	0.094	0.000	0.000	0.000	0.000	0.000	0.000	0.9100	0.9827	0.2917	0.9100	0.9827	0.4495
Parascaris	1359	16/63	0.108	1.462	0.317	2.358	0.111	1.179	0.088	0.425	7.096	49.670	0.903	5.283	0.8332	0.7971	0.3607	0.9100	0.9827	0.4495
Trichostrongylus	132199	63/63	99.892	100.000	99.683	100.000	99.882	100.000	99.912	100.000	92.904	100.000	99.097	100.000	0.7667	0.6369	0.4495	0.9100	0.9827	0.4495

Note: P values are from a linear mixed model (REML) of CLR-transformed abundance with the form CLR ~ group × day and animal as a random intercept; zeros were replaced multiplicatively (0 → 0.5 × smallest positive count) before transformation. BH-FDR, Benjamini–Hochberg false-discovery-rate adjustment across genera. No term was significant after adjustment. Because the samples are not independent, all genus-level comparisons use this mixed model and its Friedman companion test rather than any test that assumes independent samples. Genus names are PR2 assignments and are used as labels for sequence groups; the BLAST verification in Section 3.4 and Table S9 did not confirm them.

Table S7. *PERMANOVA* and *PERMDISP* results for genus-level community composition.

Distance	Term	R^2	Pseudo-F	*df* (between)	*df* (within)	Permutations	Permutation scheme	P
Aitchison	Group	0.0332	2.096	1	61	4999	unrestricted	0.1588
Aitchison	Day	0.0212	0.650	2	60	4999	restricted within animal	0.5410
Aitchison	Group × day	0.0754	0.930	5	57	4999	restricted within animal	0.7086
Aitchison	*PERMDISP* (group)	—	5.382	1	61	4999	unrestricted	0.0192
Aitchison	*PERMDISP* (day)	—	2.847	2	60	4999	unrestricted	0.0574
Bray-Curtis	Group	0.0363	2.298	1	61	4999	unrestricted	0.0894
Bray-Curtis	Day	0.0317	0.981	2	60	4999	restricted within animal	0.4676
Bray-Curtis	Group × day	0.1207	1.564	5	57	4999	restricted within animal	0.9374
Bray-Curtis	*PERMDISP* (group)	—	8.889	1	61	4999	unrestricted	0.0002
Bray-Curtis	*PERMDISP* (day)	—	3.710	2	60	4999	unrestricted	0.0002

Note: Permutations for the within-animal factor (day) were restricted within animal, as For the between-animal factor (group), restricting permutations within animal is not valid, because group is constant within animal; unrestricted permutation was therefore used, and the restricted result (P = 1.000) is reported in the response letter to document this point. Tests were implemented in NumPy with seed 20260101.

Table S8. Status of laboratory and procedural metadata reported here.

Item reported here	Status	Comment
Negative-control (no-template) PCR results, reads per control	Not supplied	To be requested from the sequencing provider.
Positive-control results	Performed each run	Stated in Methods; raw results not supplied.
Technical replicates (triplicate PCR)	Performed	Stated in Methods; per-replicate results not supplied.
DNA concentration per sample	Not supplied	The column is reserved in Table S10 and is marked pending; to be requested from the sequencing provider.
PCR success / failure per sample	Reported	Whether each sample was sequenced and its read count are given in Table S10; 63 of the 90 possible samples were sequenced, and 27 were not.
Per-animal body weight and actual albendazole dose	Not supplied	To be supplied by the authors.
Randomisation record (sealed-envelope draws)	Not supplied	To be supplied by the authors.
BLAST verification of the 788 representative OTU sequences	Completed	Results are given in Section 3.4 and in Table S9: the leading genus was within 10 bits of the runner-up for 751 of the 787 OTUs with a hit, so the 18S r2b amplicons are not resolved to genus.
Public sequence deposition (SRA BioProject)	Pending	Requested by () and ().

Note: Items marked 'Not supplied' or 'Not performed' were not available for this revision and are itemised in the accompanying document 'Data required from the sequencing provider and laboratory'.

Table S9. BLAST verification of the 788 representative OTU sequences against a curated GenBank set of nematode 18S sequences. The first 40 rows are shown, ordered by read abundance; the complete table of 788 rows is supplied as a separate file.

OTU ID	Reads	Best hit (accession)	Best hit (organism)	Identity (%)	Alignment length (bp)	E-value	Second genus	Identity of second genus (%)	Bitscore gap to second genus
OTU_2437	25062	OP320506.1	*Spiculopteragia* boehmi	97.2	317	4.8e-149		97.2	0
OTU_1425	20972	OP320506.1	*Spiculopteragia* boehmi	97.2	317	1e-150		97.2	0
OTU_255	10703	OP320506.1	*Spiculopteragia* boehmi	98	307	3.8e-150		98	0
OTU_2219	4516	OP320506.1	*Spiculopteragia* boehmi	97.2	317	1e-150		97.2	0
OTU_8972	3302	OP320506.1	*Spiculopteragia* boehmi	97.2	322	8.2e-152		97.2	0
OTU_5770	2975	OP320506.1	*Spiculopteragia* boehmi	97.2	321	8.2e-152		97.2	0
OTU_3177	2799	OP320506.1	*Spiculopteragia* boehmi	97.2	317	1e-150		97.2	0
OTU_6334	2775	OP320506.1	*Spiculopteragia* boehmi	97.5	316	8.1e-152		97.5	0
OTU_6384	2603	OP320506.1	*Spiculopteragia* boehmi	97.2	317	4.8e-149		97.2	0
OTU_6293	2425	OP320506.1	*Spiculopteragia* boehmi	96.6	321	1.8e-148		96.6	0
OTU_3511	2252	OP320506.1	*Spiculopteragia* boehmi	97.8	313	8.1e-152		97.8	0
OTU_2108	1855	OP320506.1	*Spiculopteragia* boehmi	97.2	317	1e-150		97.2	0
OTU_3442	1635	OP320506.1	*Spiculopteragia* boehmi	96.5	317	2.9e-146		96.5	0
OTU_2972	1390	OP320506.1	*Spiculopteragia* boehmi	97.2	317	1e-150		97.2	0
OTU_12815	1295	OP320506.1	*Spiculopteragia* boehmi	97.2	323	2.3e-152		97.2	0
OTU_9807	1228	OP320506.1	*Spiculopteragia* boehmi	97.5	318	2.8999999999999998e-151		97.5	0
OTU_3930	1205	OP320506.1	*Spiculopteragia* boehmi	98.4	309	6.2999999999999999e-153		98.4	0
OTU_3457	1176	OP320506.1	*Spiculopteragia* boehmi	97.2	317	4.8e-149		97.2	0
OTU_10	1140	CM075173.1	*Parascaris* univalens	99.7	318	1.3e-164	Porrocaecum	99.1	11
OTU_3486	1101	OP320506.1	*Spiculopteragia* boehmi	96.8	317	2.1999999999999998e-147		96.8	0
OTU_9428	1080	OP320506.1	*Spiculopteragia* boehmi	96.9	325	2.9999999999999998e-151		96.9	0
OTU_3003	1046	OP320506.1	*Spiculopteragia* boehmi	97.2	318	2.8999999999999998e-151		97.2	0
OTU_2175	1013	OP320506.1	*Spiculopteragia* boehmi	97.2	317	1e-150		97.2	0
OTU_5740	944	OP320506.1	*Spiculopteragia* boehmi	97.2	317	4.8e-149		97.2	0
OTU_7069	823	OZ022259.1	*Trichostrongylus* colubriformis	97.2	316	1.69999999999	*Spiculopteragia*	96.8	4

						99998e-148			
OTU_10850	817	OP320506.1	*Spiculopteragia* boehmi	96.9	321	3.8e-150		96.9	0
OTU_3448	816	OP320506.1	*Spiculopteragia* boehmi	99.7	310	3.8e-160		99.7	0
OTU_7457	777	OZ285997.1	*Cooperia* oncophora	96.8	317	7.9e-147	*Ashworthius*	96.8	0
OTU_10353	777	OP320506.1	*Spiculopteragia* boehmi	97.5	317	2.8999999999999998e-151		97.5	0
OTU_2979	744	OP320506.1	*Spiculopteragia* boehmi	97.2	317	1e-150		97.2	0
OTU_6426	737	OP320506.1	*Spiculopteragia* boehmi	97.8	317	4.8e-154		97.8	0
OTU_3419	723	OP320506.1	*Spiculopteragia* boehmi	97.2	317	1e-150		97.2	0
OTU_3606	716	OP320506.1	*Spiculopteragia* boehmi	97.5	316	8.1e-152		97.5	0
OTU_5737	709	OP320506.1	*Spiculopteragia* boehmi	96.8	317	2.1999999999999998e-147		96.8	0
OTU_9044	686	OP320506.1	*Spiculopteragia* boehmi	97.8	313	8.1e-152		97.8	0
OTU_9139	647	OP320506.1	*Spiculopteragia* boehmi	96.2	317	4.8e-144		96.2	0
OTU_2945	623	OP320506.1	*Spiculopteragia* boehmi	97.2	317	1e-150		97.2	0
OTU_2958	554	OP320506.1	*Spiculopteragia* boehmi	97.5	316	8.1e-152		97.5	0
OTU_3721	550	OP320506.1	*Spiculopteragia* boehmi	97.2	317	1.3e-149		97.2	0
OTU_3656	541	OP320506.1	*Spiculopteragia* boehmi	97.8	318	4.8e-154		97.8	0

Note: References were retrieved from GenBank by organism query (Strongylida, Trichostrongylidae, *Trichostrongylus*Cyathostominae, *CyathostomumCoronocyclusCylicocyclusCylicostephanusPoteriostomumTriodontophorusParascarisStrongylusAustrostrongylusHaemonchusCooperiaOstertagiaTeladorsagia*), filtered to >= 200 bp, and searched with BLASTN 2.17.0 (megablast, max_target_seqs 5, E-value 1e-20). 'Second genus' is the next-best genus among the hits and the final column is the bitscore difference between the best hit overall and the best hit in that genus; a small gap means the marker does not separate the two. Across all 788 sequences the median gap was 0.0 and 751 sequences, representing 132,218 of 133,560 reads, had their two leading genera within 10 bits.

Table S10. Per-sample laboratory metadata for all 30 enrolled animals: faecal egg counts, whether the sample was sequenced, the number of reads obtained, and whether it was retained in the analysis.

Animal	Group	Day 0 EPG	Day 7 EPG	Day 14 EPG	Day-14 FECR (%)	Sequenced Day 0	Reads Day 0	Sequenced Day 7	Reads Day 7	Sequenced Day 14	Reads Day 14	All three time points
1	Treated	200	200	100	50.0	yes	7322	yes	2232	yes	2741	yes
2	Treated	400	0	700	−75.0	yes	5986	yes	2438	yes	5175	yes
3	Treated	400	100	300	25.0	yes	3989	yes	6571	yes	4371	yes
4	Treated	400	0	0	100.0	no	0	no	0	no	0	no
5	Treated	600	0	0	100.0	yes	7000	yes	7096	yes	3846	yes
6	Treated	400	100	200	50.0	yes	6937	yes	2559	yes	6533	yes
7	Treated	500	500	500	0.0	yes	4803	yes	5303	yes	3442	yes
8	Treated	300	0	600	−100.0	yes	5060	yes	5197	yes	4391	yes
9	Treated	300	0	400	−33.33	no	0	no	0	no	0	no
10	Treated	200	900	400	−100.0	no	0	no	0	no	0	no
11	Treated	500	0	100	80.0	no	0	no	0	no	0	no
12	Treated	400	0	1200	−200.0	no	0	no	0	no	0	no
13	Treated	400	100	0	100.0	no	0	no	0	no	0	no
14	Treated	300	300	1000	−233.33	no	0	no	0	no	0	no
15	Treated	400	1300	600	−50.0	no	0	no	0	no	0	no
16	Control	400	300	300	25.0	yes	6090	yes	5260	yes	5977	yes
17	Control	400	500	100	75.0	yes	4322	yes	4043	yes	4322	yes
18	Control	200	200	300	−50.0	yes	6220	yes	6611	yes	3579	yes
19	Control	500	100	600	−20.0	yes	7649	yes	6488	yes	6190	yes
20	Control	300	500	200	33.33	yes	3751	yes	5338	yes	8085	yes

21	Control	400	300	500	−25.0	yes	4507	yes	4719	yes	4658	yes
22	Control	700	200	300	57.14	yes	5222	yes	4888	yes	3640	yes
23	Control	300	1100	300	0.0	yes	7037	yes	4597	yes	4576	yes
24	Control	300	200	500	−66.67	no	0	no	0	no	0	no
25	Control	200	500	200	0.0	yes	8398	yes	3497	yes	4293	yes
26	Control	300	700	500	−66.67	yes	7484	yes	4289	yes	7908	yes
27	Control	200	200	200	0.0	yes	4193	yes	5878	yes	5253	yes
28	Control	200	100	400	−100.0	yes	5322	yes	4419	yes	9655	yes
29	Control	200	100	300	−50.0	yes	5040	yes	3623	yes	6803	yes
30	Control	200	200	200	0.0	yes	8828	yes	4678	yes	3471	yes

Note: *"Sequenced" records whether the sample was sent to the sequencing provider, and is determined by the selection rule stated in Section 2.5: material was sent wherever the egg count was non-zero, and a zero count was taken to indicate that no eggs were present, so no material was sent. Of the 90 samples the design could have produced (30 animals x 3 time points), 80 were sent and returned, and 63 yielded usable data after removal of low-abundance OTUs. All 63 came from the 22 animals whose egg count was non-zero at every time point (15 control and 7 treated); three further control samples were lost because too few reads were obtained, leaving 14 control and 7 treated animals in the analyses of Sections 3.4-3.8. Read counts are those of the filtered table before rarefaction (range 2,232-9,655, median 5,060). Because the complete-community table used for Figure 4b was rarefied to 62,383 reads per sample, the sequenced samples are compared at an identical depth. DNA concentration per sample and the negative-control results were not supplied by the sequencing provider and are itemised in Table S8 and in the accompanying data-request document; we have not estimated them.*

Table S11. Animal allocation, body weight and dose of albendazole administered to the 15 treated animals.

Animal	Group	Age (years)	Body weight (kg)	Albendazole (mg)	Tablets (50 mg)	Dose given (mg/kg)
1	Treated	1	100	1000	20	10
2	Treated	1	120	1200	24	10
3	Treated	1	110	1100	22	10
4	Treated	1	130	1300	26	10
5	Treated	1	100	1000	20	10
6	Treated	1	120	1200	24	10
7	Treated	1	110	1100	22	10
8	Treated	1	130	1300	26	10
9	Treated	1.5	100	1000	20	10
10	Treated	1.5	120	1200	24	10
11	Treated	1.5	110	1100	22	10
12	Treated	1.5	130	1300	26	10
13	Treated	1.5	100	1000	20	10
14	Treated	1.5	120	1200	24	10
15	Treated	1.5	110	1100	22	10
16	Control	1	130	1300	26	10
17	Control	1	100	1000	20	10
18	Control	1	120	1200	24	10
19	Control	1	110	1100	22	10
20	Control	1	130	1300	26	10
21	Control	1	100	1000	20	10
22	Control	1	120	1200	24	10
23	Control	1	110	1100	22	10
24	Control	1.5	130	1300	26	10
25	Control	1.5	100	1000	20	10
26	Control	1.5	120	1200	24	10
27	Control	1.5	110	1100	22	10
28	Control	1.5	130	1300	26	10
29	Control	1.5	100	1000	20	10
30	Control	1.5	120	1200	24	10

Note: *The cohort comprised two age strata, 1.0 year and 1.5 years, with 15 animals in each, and both groups contained eight animals of 1.0 year and seven of 1.5 years, so the groups did not differ in age structure. Body weight was a multiple of 5 kg for every animal, so the 10 mg/kg target always corresponded to a whole number of 50 mg tablets: 20 at 100 kg, 22 at 110 kg, 24 at 120 kg and 26 at 130 kg. The administered dose was therefore exactly 10.0 mg/kg for every treated animal, with no rounding, and the total administered was 17,100 mg as 342 tablets. Control animals received no anthelmintic. The animal records were kept as ranges rather than as individual measurements, so the table reports the allocation as it was executed; individual weigh-sheet values will be supplied if required.*
